# Systematic evaluation of machine learning models for clinical risk prediction on real-world hospital datasets

**DOI:** 10.1016/j.isci.2026.115654

**Published:** 2026-04-08

**Authors:** Qingwen Wu, Ziyou Qi, Qingwei Li, Cuiping Hao, Sujuan Tang

**Affiliations:** 1 Department of Data Center, Affiliated Hospital of Jining Medical University, Jining, China; 2 Department of Neurological Intensive Care Unit, Affiliated Hospital of Jining Medical University, Jining, China; 3 Department of Spinal Surgery, Affiliated Hospital of Jining Medical University, Jining, China; 4 Department of Intensive Care Unit, Affiliated Hospital of Jining Medical University, Jining, China

**Keywords:** health sciences, medicine, bioinformatics

## Abstract

The application of machine learning in clinical medicine requires systematic evaluation across diverse modeling paradigms. We benchmarked 10 models, including classic machine learning, tabular deep learning, and automated machine learning (AutoML), across eight real-world clinical risk prediction datasets. Using a 10-time repeated 5-fold cross-validation protocol, we assessed discrimination, calibration, and clinical utility. Gradient boosting decision trees, particularly CatBoost, and the tabular foundation model TabPFN consistently demonstrated superior robustness, forming the top tier for performance. AutoGluon also exhibited strong competitiveness. In contrast, most other tabular deep learning models displayed significant instability. These findings indicate that advanced gradient boosting models and TabPFN represent premier strategies for building high-performance clinical risk prediction models, while AutoML offers a reliable alternative. This study provides crucial empirical guidance for clinicians and data scientists in selecting appropriate modeling strategies.

## Highlights

• Systematic evaluation of 10 models on 8 real-world clinical datasets
• CatBoost and TabPFN demonstrated superior discrimination and calibration
• Most tabular deep learning models displayed significant performance instability
• AutoML offers a robust, high-performance solution for non-expert users

## Graphical abstract

**Figure:**
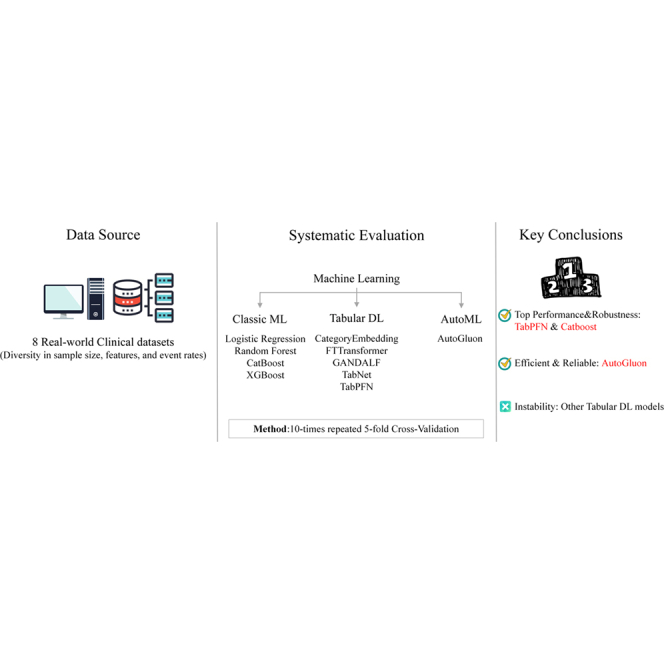

## Introduction

With the rapid proliferation of data from electronic health records (EHRs), medical imaging, genomics, and wearable devices, clinical medicine is rapidly entering a new, data-driven era. This vast amount of multimodal health data offers unprecedented opportunities for developing precise and efficient clinical decision support systems (CDSSs).^1^^,^^2^ As a core component of CDSSs, clinical risk prediction models are designed to identify individuals at high risk of future adverse events, such as disease onset, progression, or mortality, by analyzing patient data. Consequently, these models are of paramount importance for enabling early disease warning, personalized interventions, and the optimal allocation of healthcare resources.^3^^,^^4^

Over the past decade, classic machine learning (ML) algorithms have been widely applied and have demonstrated significant efficacy in the field of clinical risk prediction.^5^^,^^6^^,^^7^ Valued for its excellent interpretability and computational efficiency, logistic regression (LR) frequently serves as a baseline model in clinical research.^8^^,^^9^ Concurrently, ensemble learning models, exemplified by random forest (RF), XGBoost, and CatBoost, have exhibited strong performance in diverse prediction tasks across cardiology, sepsis, and oncology, leveraging their robust ability to model non-linear relationships and their resilience to high-dimensional features.^10^^,^^11^^,^^12^^,^^13^^,^^14^^,^^15^ However, these traditional models often rely on laborious and time-consuming manual feature engineering, and their performance ceiling is consequently constrained by the prior knowledge of domain experts.

To overcome the limitations of traditional methods, the research community has increasingly turned its attention to deep learning (DL) and automated machine learning (AutoML) models. Following its transformative success in processing unstructured data like images and text, the application of DL to structured tabular data has emerged as a new research frontier. Consequently, a series of novel DL models tailored for tabular data have been developed. For instance, the TabNet model introduces a sequential attention mechanism to mimic the decision-making process of decision trees, thereby balancing model performance with interpretability.^16^ The FTTransformer borrows from the success of Transformers in natural language processing, employing an attention mechanism to effectively capture complex interactions between features.^17^ TabPFN, a foundation model specifically designed for tabular data,^18^ utilizes an in-context learning (ICL) framework to pre-train a Transformer on millions of synthetic tabular tasks, enabling it to make predictions on new data at inference time without requiring further training.^19^ Furthermore, models such as GANDALF and CategoryEmbedding (hereafter referred to as Category) have offered various other avenues to enhance representation learning for tabular data. A key advantage of these models lies in their end-to-end learning paradigm, which allows them to automatically learn effective feature representations from raw data, thus reducing the dependency on manual feature engineering.

Meanwhile, the rise of AutoML technology provides an accessible pathway for implementing ML in clinical settings.^20^ AutoML frameworks, such as AutoGluon, integrate a suite of advanced models with automated hyperparameter tuning and model ensembling techniques to offer a high-performance, “out-of-the-box” solution for non-experts.^21^^,^^22^^,^^23^ This substantially lowers the technical barrier to entry for building and deploying high-quality prediction models, allowing clinicians and researchers to focus more on the underlying clinical questions.

Despite the proliferation of these new models and technologies, a significant knowledge gap persists: there is a lack of systematic, head-to-head comparative studies evaluating classic ML, state-of-the-art tabular DL, and AutoML frameworks on unified, real-world, and diverse clinical datasets. Most existing studies have presented comparisons on a limited scope or relied on public benchmark datasets, meaning that the generalizability and reliability of these benchmarks in authentic clinical settings remain to be validated.^24^^,^^25^^,^^26^ This absence of comprehensive benchmarks makes it challenging for researchers, when faced with a specific prediction task, to make informed trade-offs between model performance, computational cost, and practicality to select the most suitable modeling strategy.

The eight clinical tasks benchmarked in this study were specifically chosen to represent a range of high-impact medical challenges where traditional scoring systems are often inadequate. These include predicting high-mortality conditions in critical care, such as influenza-associated aspergillosis (IAA) and ICU-acquired acute kidney injury (AKI), as well as managing chronic disease burdens like postoperative osteoporotic re-fracture. Such problems are characterized by their reliance on subtle, non-linear interactions among numerous biomarkers and clinical features, making them ideal testbeds for evaluating which modern ML paradigms can provide the most reliable decision support.

To improve this limitation, the present study aims to provide evidence-based methodological guidance to clinical researchers. By benchmarking these paradigms on high-burden clinical tasks, we aim to identify which modeling strategies yield the safest and most reliable risk estimates for patient care, thereby preventing the deployment of unstable or overfitted models in clinical practice. We curated a collection of eight real-world clinical risk prediction datasets from the Affiliated Hospital of Jining Medical University, spanning various medical departments and prediction tasks and covering both general clinical information and laboratory data. Using these datasets, we conducted a comprehensive performance evaluation of three representative categories of models: (1) classic ML models (LR, RF, XGBoost, and CatBoost); (2) advanced DL models for tabular data (TabPFN, Category, TabNet, GANDALF, and FTTransformer); and (3) a leading AutoML framework (AutoGluon). Through a rigorous 10-time repeated 5-fold cross-validation scheme, we conducted an in-depth analysis of model performance across multiple dimensions, including discrimination (receiver operating characteristic [ROC] and precision-recall [PR] curves), calibration (calibration curves), and clinical utility (sensitivity, specificity, and positive/negative predictive values [PPVs/NPVs]). The objective of this study is to establish a clear benchmark for the performance of different modeling paradigms on real-world clinical data, thereby providing empirical evidence and practical guidance for researchers and data scientists in selecting and applying appropriate models for future risk prediction tasks.

## Results

In this chapter, we systematically present the performance of all models across the eight clinical risk prediction datasets.

### Performance on Dataset 1 and Dataset 2

#### Comparison of discrimination

**Figure 1:**
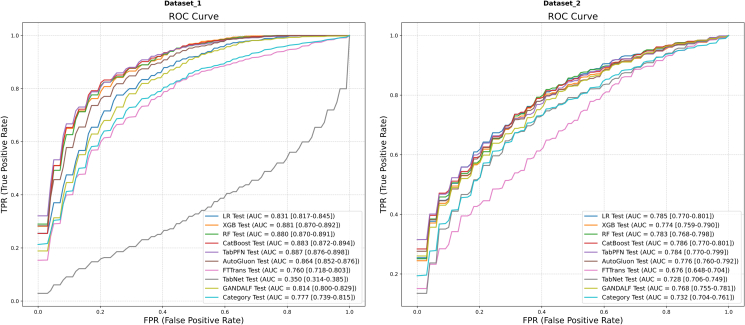
Mean ROC curves Results for Dataset 1 (left) and Dataset 2 (right) are shown. AUC values are presented with their 95% CIs.

**Figure 2:**
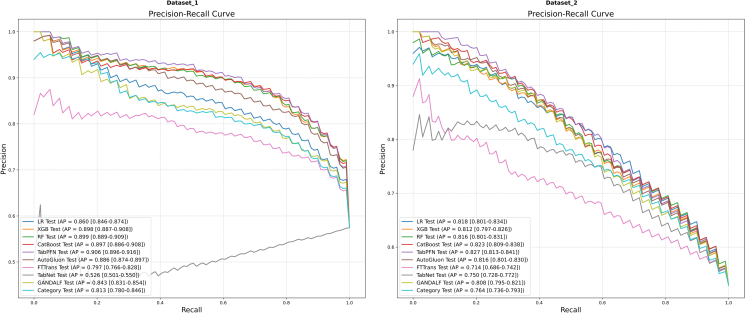
Mean PR curves Results for Dataset 1 (left) Dataset 2 (right) are shown. AP values are presented with their 95% CIs.

The mean ROC and PR curves for all models on Dataset 1 and Dataset 2 are shown in Figures 1 and 2, respectively.

On Dataset 1 (Figure 1, left; Figure 2, left), the models exhibited considerable differences in their discrimination capabilities. In terms of AUC, several models demonstrated excellent performance. Specifically, TabPFN (AUC = 0.887, 95% confidence interval [CI, 0.876, 0.898]), CatBoost (AUC = 0.883, 95% CI [0.872, 0.894]), XGBoost (AUC = 0.881, 95% CI [0.870, 0.892]), and RF (AUC = 0.880, 95% CI [0.870, 0.891]) constituted a top-performing tier with highly comparable performance. AutoGluon (AUC = 0.864) and the baseline model LR (AUC = 0.831) also delivered robust performance. In contrast, some DL models underperformed, particularly TabNet (AUC = 0.350, 95% CI [0.314, 0.385]), whose performance was substantially below the level of random chance. With respect to area under the PR curve (AP), TabPFN (AP = 0.906), RF (AP = 0.899), XGBoost (AP = 0.898), and CatBoost (AP = 0.897) delivered the most outstanding performance, outperforming the other models. TabNet also registered the lowest AP (AP = 0.526).

On Dataset 2 (Figure 1, right; Figure 2, right), a general decline in model performance was observed compared with Dataset 1. CatBoost demonstrated the best discrimination, achieving the highest AUC of 0.786 (95% CI [0.770, 0.801]). LR (AUC = 0.785) was a close second. Conversely, FTTransformer (AUC = 0.676) and TabNet (AUC = 0.728) exhibited the weakest performance on this dataset. In terms of AP, TabPFN (AP = 0.827) and CatBoost (AP = 0.823) delivered comparable and leading performance. The performance of LR (AP = 0.818) was also competitive.

#### Comparison of calibration

**Figure 3:**
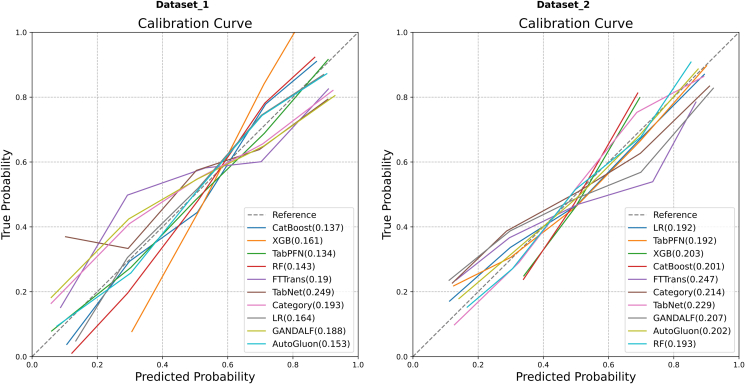
Comparison of calibration curves on Dataset 1 and Dataset 2.

The calibration curves for the models are displayed in Figure 3, where the value in parentheses is the Brier score; a lower score indicates better calibration.

On Dataset 1 (Figure 3, left), the calibration curves of most models were well-aligned with the ideal diagonal. Among them, TabPFN and CatBoost had the lowest Brier scores of 0.134 and 0.137, respectively, indicating the best calibration performance. In contrast, the calibration curve for TabNet (Brier score = 0.249) deviated substantially from the diagonal, signifying the poorest calibration.

On Dataset 2 (Figure 3, right), the calibration curves for TabPFN (Brier score = 0.192) and LR (Brier score = 0.192) were the most closely aligned with the diagonal, demonstrating superior calibration. FTTransformer had the highest Brier score (0.247), indicating the worst calibration performance.

#### Comparison of clinical utility

The clinical utility metrics, calculated based on the optimal threshold, are summarized in Table 1.

**Table 1:** Clinical utility indicators on Dataset 1 and Dataset 2

Data	Model	PPV	NPV	Sensitivity	Specificity
Data_1	LR	0.7751[0.7606, 0.7895]	0.7557[0.7359, 0.7754]	0.8366[0.8192, 0.8540]	0.6659[0.6376, 0.6941]
	XGB	0.7710[0.7612, 0.7807]	0.8004[0.7843, 0.8165]	0.8746[0.8616, 0.8877]	0.6427[0.6227, 0.6627]
	RF	0.7758[0.7674, 0.7842]	0.8065[0.7940, 0.8191]	0.8795[0.8697, 0.8893]	0.6501[0.6334, 0.6669]
	CatBoost	0.7817[0.7743, 0.7891]	0.8094[0.7990, 0.8199]	**0.8805[0.8725, 0.8885]**	0.6614[0.6469, 0.6759]
	AutoGluon	**0.7893[0.7832, 0.7954]**	0.8082[0.8000, 0.8164]	0.8771[0.8707, 0.8835]	**0.6772[0.6654, 0.6890]**
	TabPFN	0.7870[0.7803, 0.7937]	**0.8108[0.8018, 0.8199]**	0.8802[0.8732, 0.8872]	0.6717[0.6587, 0.6847]
	FTTrans	0.7379[0.7125, 0.7633]	0.6402[0.5730, 0.7074]	0.8255[0.7981, 0.8529]	0.5659[0.4955, 0.6360]
	TabNet	0.6359[0.6105, 0.6612]	0.4025[0.3402, 0.4649]	0.7920[0.7732, 0.8108]	0.3289[0.2666, 0.3911]
	GANDALF	0.6787[0.6584, 0.6990]	0.5197[0.4698, 0.5696]	0.8045[0.7882, 0.8208]	0.4317[0.3821, 0.4812]
	Category	0.6827[0.6610, 0.7045]	0.5578[0.5159, 0.5997]	0.7984[0.7772, 0.8197]	0.4795[0.4367, 0.5223]
Data_2	LR	0.7135[0.6984, 0.7286]	**0.6927[0.6772, 0.7082]**	0.7341[0.7142, 0.7541]	0.6630[0.6383, 0.6877]
	XGB	0.7109[0.7007, 0.7212]	0.6926[0.6808, 0.7044]	0.7344[0.7195, 0.7494]	0.6592[0.6417, 0.6768]
	RF	0.7140[0.7057, 0.7223]	0.6897[0.6802, 0.6993]	0.7276[0.7153, 0.7399]	0.6678[0.6540, 0.6817]
	CatBoost	0.7158[0.7085, 0.7230]	0.6892[0.6808, 0.6977]	0.7252[0.7145, 0.7359]	0.6721[0.6602, 0.6839]
	AutoGluon	0.7148[0.7087, 0.7208]	0.6876[0.6807, 0.6944]	0.7229[0.7137, 0.7321]	0.6703[0.6598, 0.6808]
	TabPFN	**0.7155[0.7091, 0.7219]**	0.6870[0.6795, 0.6945]	0.7218[0.7121, 0.7314]	**0.6734[0.6630, 0.6838]**
	FTTrans	0.6224[0.5937, 0.6511]	0.3378[0.2431, 0.4324]	**0.8470[0.7990, 0.8951]**	0.3390[0.2428, 0.4353]
	TabNet	0.6297[0.6110, 0.6484]	0.5236[0.4617, 0.5855]	0.8325[0.8002, 0.8648]	0.3856[0.3257, 0.4455]
	GANDALF	0.6588[0.6439, 0.6738]	0.5694[0.5265, 0.6122]	0.7812[0.7554, 0.8071]	0.4870[0.4399, 0.5341]
	Category	0.6667[0.6519, 0.6815]	0.5697[0.5335, 0.6059]	0.7619[0.7377, 0.7861]	0.5195[0.4788, 0.5602]

Bold entries represent the best performance achieved for each respective metric.

On Dataset 1, different models excelled on different metrics, highlighting a trade-off in performance. AutoGluon achieved the best performance in terms of PPV (0.7893) and specificity (0.6772). TabPFN obtained the highest NPV (0.8108). CatBoost demonstrated the highest sensitivity (0.8805), indicating its superior ability to identify positive cases. Notably, TabNet’s specificity was only 0.3289, implying a very high false-positive rate, which is consistent with its extremely low AUC result.

On Dataset 2, similar performance trade-offs among models were observed. TabPFN ranked first on both PPV (0.7155) and specificity (0.6734), demonstrating a strong overall performance. The baseline model LR achieved the highest NPV (0.6927). A noteworthy finding is that although FTTransformer had a relatively low overall discrimination (AUC), its sensitivity (0.8470) was the highest among all models. This suggests that at its optimal threshold, it could minimize missed diagnoses to the greatest extent, but this was achieved at the cost of a very low specificity (0.3390).

### Performance on Dataset 3 and Dataset 4

#### Comparison of discrimination

**Figure 4:**
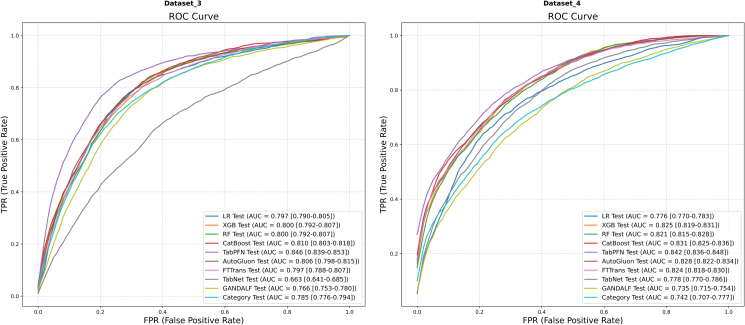
Mean ROC curves Results for Dataset 3 (left) and Dataset 4 (right) are shown. AUC values are presented with their 95% CIs.

**Figure 5:**
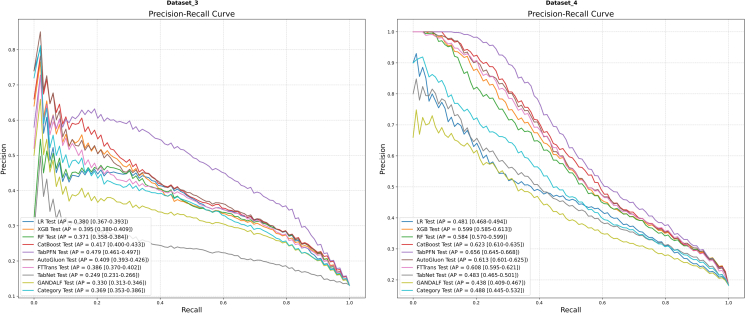
Mean PR curves Results for Dataset 3 (left) and Dataset 4 (right) are shown. AP values are presented with their 95% CIs.

The mean ROC and PR curves for all models on Dataset 3 and Dataset 4 are depicted in Figures 4 and 5, respectively.

On Dataset 3 (Figure 4, left; Figure 5, left), TabPFN demonstrated a clear superiority in discrimination. It achieved the highest AUC of 0.846 (95% CI [0.833, 0.858]) and the highest AP of 0.479 (95% CI [0.461, 0.497]), both of which were markedly better than those of the other models. CatBoost (AUC = 0.810) and AutoGluon (AUC = 0.806) delivered robust performance, forming the second tier of performers. Notably, the low overall AP values for this dataset suggest a particularly challenging prediction task.

On Dataset 4 (Figure 4, right; Figure 5, right), TabPFN (AUC = 0.842) again secured the best discrimination performance. However, the performance of CatBoost (AUC = 0.831), AutoGluon (AUC = 0.828), and XGBoost (AUC = 0.825) was highly comparable, forming a competitive top tier. In terms of AP, TabPFN (AP = 0.656) remained the leader, while CatBoost (AP = 0.623) and AutoGluon (AP = 0.631) also delivered excellent results.

#### Comparison of calibration

**Figure 6:**
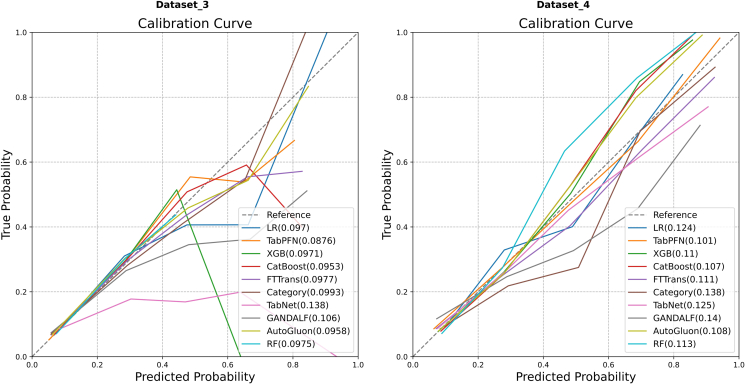
Comparison of calibration curves on Dataset 3 and Dataset 4.

The calibration performance is illustrated in Figure 6. On Dataset 3 (Figure 6, left), TabPFN exhibited the best calibration, registering the lowest Brier score (0.0876) among all models. CatBoost (Brier score = 0.0953), AutoGluon (Brier score = 0.0958), and RF (Brier score = 0.0975) also displayed good calibration, with their curves closely aligning with the ideal diagonal. On Dataset 4 (Figure 6, right), TabPFN (Brier score = 0.101) once again demonstrated its superior calibration, with the lowest Brier score. The performance of CatBoost (Brier score = 0.107) and AutoGluon (Brier score = 0.108) followed closely, proving to be highly competitive as well.

#### Comparison of clinical utility

The clinical utility metrics are summarized in Table 2. On Dataset 3, the trade-off in performance was particularly pronounced. Despite its moderate discrimination, FTTransformer achieved the highest PPV (0.4931). AutoGluon recorded the highest NPV (0.8775). Notably, the sensitivity for all models was extremely low, with GANDALF registering the highest value at just 0.1000. Conversely, RF obtained a near-perfect specificity (0.9942). This indicates a general tendency for models on this dataset to classify samples as negative to ensure high specificity, but at the cost of a high risk of missing positive cases. On Dataset 4, AutoGluon demonstrated a balanced advantage, securing the highest PPV (0.7810) and specificity (0.9816). Meanwhile, FTTransformer once again led, with the highest sensitivity (0.3512) and NPV (0.8704), continuing its trend of prioritizing high recall to avoid missed diagnoses.

**Table 2:** Clinical utility indicators on Dataset 3 and Dataset 4

Data	Model	PPV	NPV	Sensitivity	Specificity
Data_3	LR	0.4191[0.3615, 0.4767]	0.8751[0.8739, 0.8762]	0.0653[0.0552, 0.0754]	0.9863[0.9841, 0.9885]
	XGB	0.3502[0.2884, 0.4120]	0.8727[0.8718, 0.8735]	0.0390[0.0314, 0.0466]	0.9921[0.9905, 0.9938]
	RF	0.2801[0.2274, 0.3329]	0.8716[0.8709, 0.8722]	0.0276[0.0218, 0.0334]	**0.9942[0.9929, 0.9954]**
	CatBoost	0.3586[0.3129, 0.4043]	0.8737[0.8729, 0.8745]	0.0470[0.0400, 0.0541]	0.9928[0.9917, 0.9939]
	AutoGluon	0.4323[0.3977, 0.4669]	**0.8775[0.8766, 0.8785]**	0.0826[0.0737, 0.0914]	0.9894[0.9882, 0.9906]
	TabPFN	0.4084[0.3693, 0.4476]	0.8769[0.8759, 0.8780]	0.0766[0.0669, 0.0864]	0.9903[0.9891, 0.9915]
	FTTrans	**0.4931[0.4209, 0.5654]**	0.8768[0.8749, 0.8787]	0.0780[0.0602, 0.0958]	0.9876[0.9844, 0.9909]
	TabNet	0.3266[0.2712, 0.3820]	0.8753[0.8739, 0.8767]	0.0861[0.0674, 0.1048]	0.9644[0.9528, 0.9760]
	GANDALF	0.3339[0.2911, 0.3767]	0.8771[0.8756, 0.8786]	**0.1000[0.0833, 0.1167]**	0.9648[0.9565, 0.9730]
	Category	0.3503[0.3118, 0.3887]	0.8764[0.8752, 0.8776]	0.0885[0.0751, 0.1018]	0.9712[0.9648, 0.9776]
Data_4	LR	0.5940[0.5682, 0.6198]	0.8472[0.8448, 0.8497]	0.2195[0.2040, 0.2350]	0.9663[0.9632, 0.9694]
	XGB	0.6891[0.6638, 0.7144]	0.8543[0.8520, 0.8565]	0.2559[0.2430, 0.2689]	0.9741[0.9717, 0.9765]
	RF	0.7524[0.7278, 0.7770]	0.8502[0.8484, 0.8521]	0.2258[0.2141, 0.2374]	0.9809[0.9786, 0.9832]
	CatBoost	0.7673[0.7475, 0.7871]	0.8538[0.8520, 0.8556]	0.2467[0.2359, 0.2575]	0.9815[0.9797, 0.9833]
	AutoGluon	**0.7810[0.7667, 0.7952]**	0.8588[0.8572, 0.8604]	0.2764[0.2667, 0.2860]	**0.9816[0.9802, 0.9829]**
	TabPFN	0.7754[0.7589, 0.7918]	0.8578[0.8560, 0.8596]	0.2708[0.2598, 0.2817]	0.9812[0.9797, 0.9827]
	FTTrans	0.7462[0.7156, 0.7769]	**0.8704[0.8657, 0.8751]**	**0.3512[0.3215, 0.3810]**	0.9683[0.9617, 0.9750]
	TabNet	0.6908[0.6616, 0.7200]	0.8581[0.8544, 0.8618]	0.2784[0.2552, 0.3017]	0.9700[0.9658, 0.9743]
	GANDALF	0.6336[0.6021, 0.6651]	0.8560[0.8525, 0.8594]	0.2711[0.2484, 0.2937]	0.9596[0.9516, 0.9676]
	Category	0.5874[0.5505, 0.6244]	0.8487[0.8418, 0.8556]	0.2645[0.2396, 0.2894]	0.9470[0.9274, 0.9666]

Bold entries represent the best performance achieved for each respective metric.

### Performance on Dataset 5 and Dataset 6

#### Comparison of discrimination

**Figure 7:**
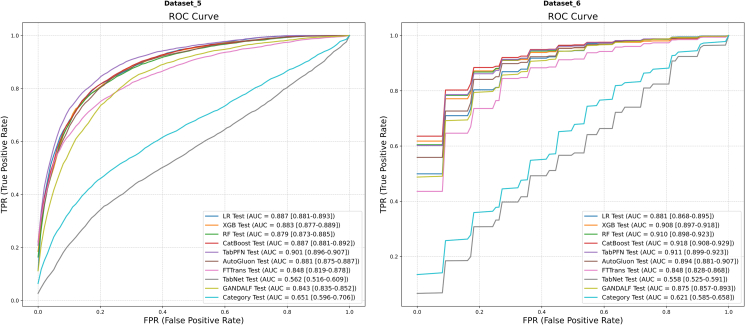
Mean ROC curves Results for Dataset 5 (left) and Dataset 6 (right) are shown. AUC values are presented with their 95% CIs.

**Figure 8:**
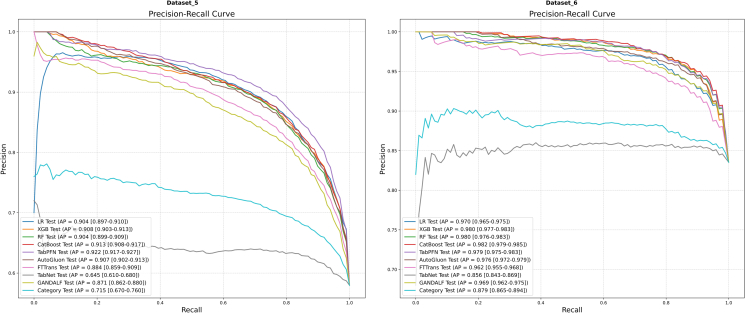
Mean PR curves Results for Dataset 5 (left) and Dataset 6 (right) are shown. AP values are presented with their 95% CIs.

The mean ROC and PR curves for all models on Dataset 5 and Dataset 6 are presented in Figures 7 and 8, respectively.

On Dataset 5 (Figure 7, left), the models exhibited broadly high discrimination capabilities. The performance of TabPFN (AUC = 0.901) was particularly notable, holding a slight edge over LR (AUC = 0.887) and CatBoost (AUC = 0.887). These three models collectively formed the top-performance tier. In terms of AP (Figure 8, left), TabPFN (AP = 0.922) again achieved the optimal value, with CatBoost (AP = 0.913) and XGBoost (AP = 0.908) following closely, with minimal difference in performance.

Moving to Dataset 6 (Figure 7, right), the models’ discrimination performance ascended to an even higher level. The ROC curves displayed a unique step-like pattern, which may be attributable to the presence of highly discriminative features within the dataset. CatBoost (AUC = 0.918) and RF (AUC = 0.910) delivered comparable, top-ranking performance. With respect to AP (Figure 8, right), multiple models demonstrated near-perfect performance. The average precision scores for XGBoost (AP = 0.980), CatBoost (AP = 0.982), and TabPFN (AP = 0.979) were all extremely close to 1.0, indicating their exceptional ability to identify positive samples on this dataset.

#### Comparison of calibration

**Figure 9:**
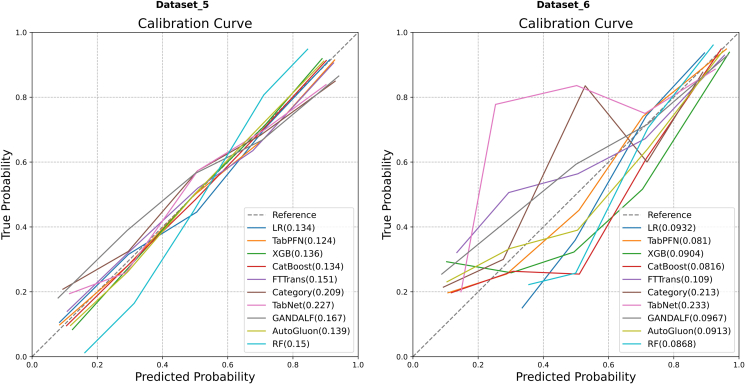
Comparison of calibration curves on Dataset 5 and Dataset 6.

The calibration curves for the models are shown in Figure 9. The calibration assessment revealed notable differences among the models. On Dataset 5 (Figure 9, left), TabPFN exhibited optimal calibration properties, evidenced by the lowest Brier score (0.124). The calibration of LR (Brier score = 0.134) and CatBoost (Brier score = 0.134) was also found to be reliable. On Dataset 6 (Figure 9, right), Brier scores were generally low across all models. Among them, TabPFN (Brier score = 0.0810) likewise demonstrated an excellent level of calibration.

#### Comparison of clinical utility

The clinical utility metrics, calculated based on the optimal threshold, are summarized in Table 3. The analysis of these metrics highlighted the strategic preferences of the models across different datasets. On Dataset 5, TabPFN demonstrated a comprehensive advantage, simultaneously topping the leaderboards for four key metrics—PPV (0.8226), NPV (0.7946), sensitivity (0.8599), and specificity (0.7427)—signifying its outstanding overall performance.

**Table 3:** Statistical test results of AUC performance differences between TabPFN, AutoGluon, and other models (*p* values)

	Dataset	FTT	GAND	Categ	RF	AutoG	LR	CatB	XGB	TabN
**T a** **b** **P**	Data_1	*p* < 0.01	*p* < 0.01	*p* < 0.01	*p* < 0.01	*p* < 0.01	*p* < 0.01	***p* = 0.05**	*p* < 0.01	*p* < 0.01
	Data_2	*p* < 0.01	*p* < 0.01	*p* < 0.01	***p* = 0.36**	*p* < 0.01	***p* = 0.35**	***p* = 0.84**	*p* < 0.01	*p* < 0.01
	Data_3	*p* < 0.01	*p* < 0.01	*p* < 0.01	*p* < 0.01	*p* < 0.01	*p* < 0.01	*p* < 0.01	*p* < 0.01	*p* < 0.01
	Data_4	*p* < 0.01	*p* < 0.01	*p* < 0.01	*p* < 0.01	*p* < 0.01	*p* < 0.01	*p* < 0.01	*p* < 0.01	*p* < 0.01
	Data_5	*p* < 0.01	*p* < 0.01	*p* < 0.01	*p* < 0.01	*p* < 0.01	*p* < 0.01	*p* < 0.01	*p* < 0.01	*p* < 0.01
	Data_6	*p* < 0.01	*p* < 0.01	*p* < 0.01	***p* = 0.32**	*p* < 0.01	*p* < 0.01	*p* < 0.01	***p* = 0.96**	*p* < 0.01
	Data_7	*p* < 0.01	*p* < 0.01	*p* < 0.01	***p* = 0.10**	***p* = 0.08**	*p* < 0.01	***p* = 0.21**	*p* < 0.01	*p* < 0.01
	Data_8	*p* < 0.01	*p* < 0.01	*p* < 0.01	*p* < 0.01	*p* < 0.01	*p* < 0.01	***p* = 0.57**	*p* < 0.01	*p* < 0.01
	**Dataset**	**FTT**	**GAND**	**Categ**	**RF**	**TabP**	**LR**	**CatB**	**XGB**	**TabN**
**A u** **t** **o** **G**	Data_1	*p* < 0.01	*p* < 0.01	*p* < 0.01	*p* < 0.01	*p* < 0.01	*p* < 0.01	*p* < 0.01	*p* < 0.01	*p* < 0.01
	Data_2	*p* < 0.01	***p* = 0.23**	*p* < 0.01	*p* < 0.01	*p* < 0.01	*p* < 0.01	*p* < 0.01	*p* < 0.01	*p* < 0.01
	Data_3	*p* < 0.01	*p* < 0.01	*p* < 0.01	***p* = 0.12**	*p* < 0.01	***p* = 0.13**	*p* < 0.01	***p* = 0.09**	*p* < 0.01
	Data_4	*p* < 0.01	*p* < 0.01	*p* < 0.01	*p* < 0.01	*p* < 0.01	*p* < 0.01	***p* = 0.01**	***p* = 0.51**	*p* < 0.01
	Data_5	*p* < 0.01	*p* < 0.01	*p* < 0.01	***p* = 0.36**	*p* < 0.01	*p* < 0.01	*p* < 0.01	*p* < 0.01	*p* < 0.01
	Data_6	*p* < 0.01	***p* = 0.04**	*p* < 0.01	*p* < 0.01	*p* < 0.01	***p* = 0.59**	*p* < 0.01	*p* < 0.01	*p* < 0.01
	Data_7	*p* < 0.01	*p* < 0.01	*p* < 0.01	***p* = 0.75**	***p* = 0.08**	*p* < 0.01	***p* = 0.44**	***p* = 0.20**	*p* < 0.01
	Data_8	*p* < 0.01	*p* < 0.01	*p* < 0.01	***p* = 0.01**	*p* < 0.01	*p* < 0.01	*p* < 0.01	*p* < 0.01	*p* < 0.01

TabP refers to TabPFN; AutoG refers to AutoGluon; FTT refers to FTTransformer; GAND refers to GANDALF; Categ refers to CategoryEmbedding; CatB refers to CatBoost; and TabN refers to TabNet.

Bold entries indicate no statistically significant difference (p ≥ 0.05) between the compared groups.

The results for Dataset 6 revealed a pronounced performance trade-off. The baseline model LR displayed remarkable sensitivity (0.9792), identifying nearly all positive cases. This, however, came at the cost of an extremely low specificity (0.3804), implying a very high false-positive rate. In stark contrast, FTTransformer adopted the opposite strategy, achieving the highest specificity (0.4714) at the expense of a lower sensitivity. Meanwhile, TabPFN excelled in terms of PPV (0.8969), indicating a high degree of reliability in its positive predictions.

### Performance on Dataset 7 and Dataset 8

#### Comparison of discrimination

**Figure 10:**
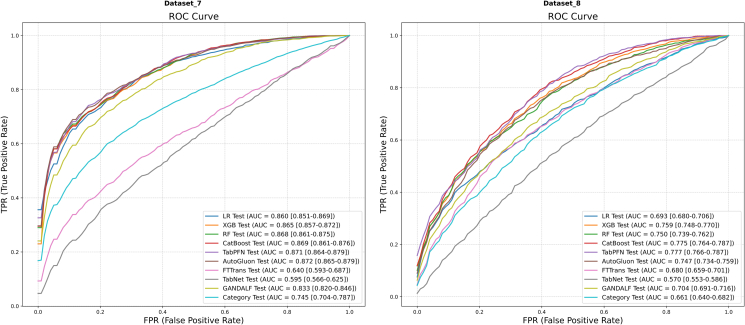
Mean ROC curves Results for Dataset 7 (left) and Dataset 8 (right) are shown. AUC values are presented with their 95% CIs.

**Figure 11:**
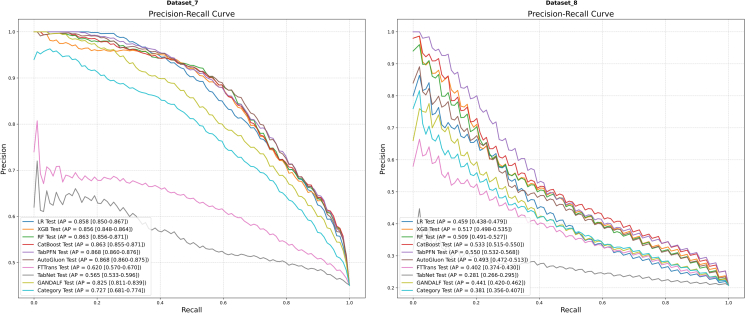
Mean PR curves Results for Dataset 7 (left) and Dataset 8 (right) are shown. AP values are presented with their 95% CIs.

The mean ROC and PR curves for all models on the final two datasets, Dataset 7 and Dataset 8, are presented in Figures 10 and 11, respectively.

In the context of Dataset 7 (Figure 10, left), AutoGluon (AUC = 0.872) and TabPFN (AUC = 0.871) emerged as the top-performing models in terms of discrimination. CatBoost (AUC = 0.869) and RF (AUC = 0.868) also demonstrated strong, competitive performance. An analysis of the PR curves (Figure 11, left) revealed a similar competitive landscape, with TabPFN (AP = 0.868) and AutoGluon (AP = 0.868) holding a slight advantage.

On Dataset 8 (Figure 10, right), a general decline in the overall discrimination ability of all models was observed, reflecting the inherent difficulty of this prediction task. Against this backdrop, TabPFN (AUC = 0.777) once again proved its robustness by achieving the highest AUC value. CatBoost (AUC = 0.775) also delivered a commendable result. The PR curves (Figure 11, right) further underscored the challenging nature of this dataset, with TabPFN (AP = 0.550) and CatBoost (AP = 0.533) outperforming the other models.

#### Comparison of calibration

**Figure 12:**
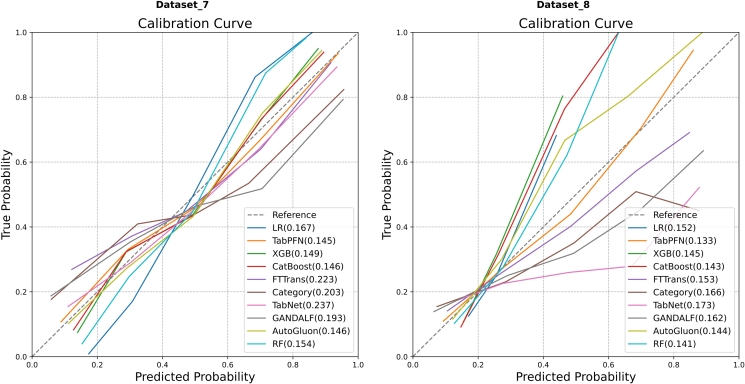
Comparison of calibration curves on Dataset 7 and Dataset 8.

The calibration curves for the models are displayed in Figure 12. Regarding predictive calibration on Dataset 7 (Figure 12, left), TabPFN (Brier score = 0.145), CatBoost (Brier score = 0.146), and AutoGluon (Brier score = 0.146) collectively demonstrated the best calibration performance, with nearly identical Brier scores. For Dataset 8 (Figure 12, right), TabPFN (Brier score = 0.133) again exhibited superior calibration properties, with its Brier score being markedly lower than those of most of its competitors. RF (Brier score = 0.141) and AutoGluon (Brier score = 0.144) also generated well-calibrated probability predictions.

#### Comparison of clinical utility

The clinical utility metrics, calculated based on the optimal threshold, are summarized in Table 4. On Dataset 7, RF excelled in precision-oriented metrics, achieving the highest PPV (0.7994) and specificity (0.8558). In contrast, AutoGluon led in recall-oriented metrics, obtaining the highest sensitivity (0.7034) and NPV (0.7794).

**Table 4:** Clinical utility indicators on Dataset 7 and Dataset 8

Data	Model	PPV	NPV	Sensitivity	Specificity
Data_7	LR	0.7891[0.7767, 0.8014]	0.7748[0.7657, 0.7840]	0.6990[0.6838, 0.7143]	0.8452[0.8338, 0.8566]
	XGB	0.7980[0.7893, 0.8067]	0.7735[0.7671, 0.7798]	0.6930[0.6819, 0.7042]	0.8543[0.8462, 0.8624]
	RF	**0.7994[0.7926, 0.8061]**	0.7735[0.7684, 0.7786]	0.6925[0.6834, 0.7016]	**0.8558[0.8494, 0.8621]**
	CatBoost	0.7974[0.7918, 0.8031]	0.7741[0.7696, 0.7785]	0.6941[0.6862, 0.7020]	0.8539[0.8485, 0.8592]
	AutoGluon	0.7984[0.7937, 0.8031]	**0.7794[0.7756, 0.7832]**	**0.7034[0.6967, 0.7101]**	0.8527[0.8483, 0.8572]
	TabPFN	0.7986[0.7935, 0.8036]	0.7769[0.7728, 0.7810]	0.6988[0.6917, 0.7060]	0.8540[0.8492, 0.8587]
	FTTrans	0.5529[0.4869, 0.6189]	0.6309[0.5924, 0.6693]	0.3790[0.2957, 0.4623]	0.8415[0.8021, 0.8810]
	TabNet	0.5685[0.5327, 0.6044]	0.6161[0.5948, 0.6375]	0.3458[0.2967, 0.3950]	0.8333[0.8100, 0.8566]
	GANDALF	0.6187[0.5895, 0.6479]	0.6673[0.6480, 0.6866]	0.4631[0.4181, 0.5081]	0.8124[0.7926, 0.8321]
	Category	0.6150[0.5863, 0.6438]	0.6694[0.6493, 0.6894]	0.4975[0.4576, 0.5373]	0.7993[0.7771, 0.8216]
Data_8	LR	0.1000[0.0168, 0.1832]	0.7926[0.7919, 0.7933]	0.0030[0.0005, 0.0056]	0.9997[0.9992, 1.0001]
	XGB	0.2900[0.2021, 0.3779]	0.7944[0.7935, 0.7953]	0.0140[0.0090, 0.0190]	**0.9997[0.9994, 1.0000]**
	RF	0.4654[0.3914, 0.5394]	0.7985[0.7971, 0.7998]	0.0392[0.0314, 0.0471]	0.9981[0.9974, 0.9988]
	CatBoost	0.5549[0.4905, 0.6193]	0.7987[0.7976, 0.7998]	0.0404[0.0342, 0.0467]	0.9984[0.9979, 0.9990]
	AutoGluon	**0.6101[0.5628, 0.6574]**	0.8052[0.8034, 0.8069]	0.0814[0.0708, 0.0919]	0.9937[0.9923, 0.9950]
	TabPFN	0.5884[0.5357, 0.6411]	0.8055[0.8035, 0.8076]	0.0834[0.0711, 0.0958]	0.9934[0.9918, 0.9950]
	FTTrans	0.4828[0.4056, 0.5601]	**0.8145[0.8091, 0.8200]**	0.1587[0.1244, 0.1931]	0.9642[0.9553, 0.9730]
	TabNet	0.3334[0.2741, 0.3927]	0.7887[0.7663, 0.8111]	0.1146[0.0812, 0.1480]	0.9534[0.9262, 0.9807]
	GANDALF	0.3833[0.3409, 0.4257]	0.8032[0.7878, 0.8185]	0.1757[0.1479, 0.2035]	0.9389[0.9200, 0.9578]
	Category	0.3886[0.3529, 0.4243]	0.8073[0.7956, 0.8189]	**0.1839[0.1608, 0.2069]**	0.9380[0.9233, 0.9526]

Bold entries represent the best performance achieved for each respective metric.

Dataset 8 exhibited an extreme performance trade-off similar to those observed on Dataset 3 and Dataset 6. AutoGluon ranked first in terms of PPV (0.6101). FTTransformer performed best in ensuring the reliability of negative predictions, achieving the highest NPV (0.8145). The Category model obtained the highest sensitivity, though this value was only 0.1839, highlighting the universal difficulty in identifying positive cases. In stark contrast, XGBoost achieved a near-perfect specificity (0.9997), implying an extremely low false-positive rate, but this was achieved at the cost of sacrificing almost all of its sensitivity (0.0140).

### Performance on a public dataset

The performance hierarchy observed on our private clinical datasets was largely preserved on Pima Indians Diabetes Database (detailed in Methods S1 and Data S2). TabPFN achieved the highest AUC (0.8407), followed closely by CatBoost (0.8335), confirming their status as top-tier performers for clinical tabular data. This external validation supported the generalizability of our main conclusions. Classic ensemble methods (RF, XGB, and CatBoost) and LR demonstrated robust performance (AUC = 0.818–0.830), with minimal performance degradation compared with our private datasets. This suggested that these models are resilient to dataset shifts between different clinical populations. AutoGluon achieved competitive results (AUC = 0.8253), ranking fifth overall and showing no significant performance gap with top-tier models. This validates its utility as an accessible “out-of-the-box” solution for clinical researchers.

Consistent with our primary analysis, most DL models exhibited significant instability. FTTransformer and TabNet substantially underperformed traditional methods (AUC < 0.60), with TabNet failing to exceed random chance (AUC = 0.4232); GANDALF showed moderate performance (AUC = 0.8113) but did not surpass gradient boosting models. Only TabPFN maintained competitive performance among DL approaches.

### Statistical significance analysis

To determine whether the performance differences observed between models in the preceding sections were attributable to random chance, we conducted pairwise comparisons of AUC values, using the Wilcoxon signed-rank test with Bonferroni correction. The resulting *p* values are summarized in Table 5. These *p* values, interpreted in conjunction with the absolute AUC values presented earlier (Figures 1, 4, 7, and 10), serve to establish whether a performance advantage or deficit is statistically significant.

**Table 5:** Clinical utility indicators on Dataset 5 and Dataset 6

Data	Model	PPV	NPV	Sensitivity	Specificity
Data_5	LR	0.8164[0.8089, 0.8240]	0.7915[0.7822, 0.8008]	0.8595[0.8519, 0.8671]	0.7319[0.7182, 0.7457]
	XGB	0.8192[0.8139, 0.8245]	0.7885[0.7815, 0.7954]	0.8555[0.8497, 0.8614]	0.7382[0.7289, 0.7476]
	RF	0.8169[0.8124, 0.8213]	0.7894[0.7836, 0.7953]	0.8573[0.8524, 0.8622]	0.7335[0.7255, 0.7414]
	CatBoost	0.8187[0.8148, 0.8225]	0.7908[0.7858, 0.7959]	0.8578[0.8536, 0.8621]	0.7365[0.7297, 0.7433]
	AutoGluon	0.8219[0.8187, 0.8251]	0.7937[0.7895, 0.7979]	0.8593[0.8558, 0.8628]	0.7417[0.7362, 0.7473]
	TabPFN	**0.8226[0.8191, 0.8261]**	**0.7946[0.7900, 0.7992]**	**0.8599[0.8560, 0.8637]**	**0.7427[0.7365, 0.7489]**
	FTTrans	0.7873[0.7506, 0.8241]	0.7165[0.6630, 0.7700]	0.8301[0.7931, 0.8671]	0.6986[0.6441, 0.7531]
	TabNet	0.7074[0.6796, 0.7353]	0.5924[0.5467, 0.6380]	0.7870[0.7586, 0.8154]	0.5340[0.4796, 0.5884]
	GANDALF	0.7405[0.7200, 0.7609]	0.6367[0.6042, 0.6692]	0.7884[0.7680, 0.8087]	0.6006[0.5605, 0.6407]
	Category	0.7210[0.7016, 0.7404]	0.6079[0.5770, 0.6389]	0.7762[0.7563, 0.7961]	0.5731[0.5373, 0.6088]
Data_6	LR	0.8895[0.8838, 0.8953]	**0.7990[0.7542, 0.8438]**	**0.9792[0.9739, 0.9845]**	0.3804[0.3444, 0.4163]
	XGB	0.8931[0.8890, 0.8973]	0.7636[0.7304, 0.7967]	0.9716[0.9668, 0.9764]	0.4071[0.3812, 0.4330]
	RF	0.8906[0.8871, 0.8941]	0.7672[0.7403, 0.7941]	0.9735[0.9698, 0.9771]	0.3899[0.3681, 0.4116]
	CatBoost	0.8922[0.8893, 0.8952]	0.7707[0.7483, 0.7930]	0.9737[0.9706, 0.9768]	0.4000[0.3811, 0.4189]
	AutoGluon	0.8964[0.8936, 0.8991]	0.7586[0.7401, 0.7771]	0.9698[0.9669, 0.9728]	0.4262[0.4089, 0.4435]
	TabPFN	**0.8969[0.8940, 0.8999]**	0.7680[0.7487, 0.7874]	0.9710[0.9680, 0.9741]	0.4293[0.4107, 0.4479]
	FTTrans	0.8814[0.8458, 0.9169]	0.6208[0.5776, 0.6639]	0.9240[0.8864, 0.9616]	**0.4714[0.4258, 0.5170]**
	TabNet	0.8680[0.8496, 0.8863]	0.4622[0.4151, 0.5092]	0.8901[0.8640, 0.9162]	0.3634[0.3234, 0.4033]
	GANDALF	0.8819[0.8691, 0.8947]	0.5413[0.5026, 0.5801]	0.9091[0.8908, 0.9275]	0.4149[0.3826, 0.4471]
	Category	0.8734[0.8634, 0.8833]	0.4670[0.4285, 0.5055]	0.9237[0.9093, 0.9380]	0.3397[0.3080, 0.3715]

Bold entries represent the best performance achieved for each respective metric.

#### Comparison of performance differences between TabPFN and other models

In combination with the AUC values observed in previous sections, *p* values, shown in Table 5, confirmed the statistical significance of TabPFN’s superior performance on multiple datasets. For instance, on Dataset 3, Dataset 4, and Dataset 5, where TabPFN achieved the highest AUC, its performance was found to be statistically superior to those of all other models (all *p* < 0.01). This indicated that TabPFN’s performance advantage on these datasets was not coincidental.

However, in cases of comparable performance, the statistical tests accurately reflected this parity. On Dataset 2, for example, the AUC value of TabPFN (0.784) was very close to those of CatBoost (0.786) and LR (0.785), and the statistical tests confirmed that these differences were not significant (*p* > 0.05). Similarly, on Dataset 6, there was no statistically significant difference between the AUC values of TabPFN (0.911) and XGBoost (0.908) (*p* = 0.96). This suggested that, in these scenarios, the performance of TabPFN was on par with these top-tier ensemble learning models.

#### Comparison of performance differences between AutoGluon and other models

The competitive performance of the AutoML framework, AutoGluon, was also statistically substantiated. On Dataset 1, AutoGluon’s AUC (0.864) was higher than that of LR (0.831), and this advantage was shown to be significant (*p* < 0.01). On Dataset 8, however, AutoGluon’s AUC (0.747) was not the highest and did not differ significantly from that of RF (0.750) (*p* = 0.014) but was significantly superiority to that of LR (0.693) (*p* < 0.01).

Conversely, the *p* values also reflected instances where AutoGluon’s performance was on par with or slightly below that of other models. For example, on Dataset 7, there were no statistically significant differences between AutoGluon (AUC = 0.872) and RF (AUC = 0.868, *p* = 0.75) or CatBoost (AUC = 0.889, *p* = 0.44). Notably, across all datasets, the superiority of AutoGluon’s AUC compared with the DL models FTTransformer, Category, and TabNet was almost invariably statistically significant.

## Discussion

This study conducted a systematic, head-to-head evaluation of classic ML models, state-of-the-art tabular DL models, and an AutoML framework across eight real-world clinical risk prediction datasets (detailed in Tables 6 and 7). Our findings provide a comprehensive benchmark for the performance of these different modeling paradigms in an authentic clinical setting. Overall, our core findings are 4-fold: (1) The emerging DL model TabPFN and top-tier gradient boosting decision tree (GBDT) models, particularly CatBoost, consistently delivered superior and robust performance, collectively forming the highest-performing tier. (2) With the exception of TabPFN, most of the tested tabular DL models exhibited significant instability, with performance fluctuating dramatically across datasets and sometimes falling short of simple baseline models. (3) The AutoML framework AutoGluon proved to be a powerful and highly competitive “out-of-the-box” solution with strong generalization capabilities. (4) The analysis of clinical utility metrics revealed inherent strategic trade-offs among the models, underscoring the importance of selecting a model based on specific clinical needs.

**Table 6:** Summary of dataset characteristics

Data	Prediction task	Sample	Positive event rate	Variables	Composition of variables
Dataset_1	CS	289	0.57	17	laboratory test indices
Dataset_2	CS	310	0.53	16	cardiac parameter indices from transthoracic echocardiography
Dataset_3	Re-fracture	1741	0.13	14	body mass index triglyceride-glucose index
Dataset_4	Re-fracture	1730	0.18	8	bone metabolism indices
Dataset_5	Adenomyosis	1192	0.58	27	laboratory test indices
Dataset_6	IAA	340	0.84	54	combination drug, laboratory test indices
Dataset_7	AKI in ICU	741	0.45	61	laboratory test indices
Dataset_8	DVT in ICU	789	0.21	86	laboratory test indices

CS, cryptogenic stroke; IAA, influenza-associated aspergillosis; AKI, acute kidney injury; DVT, deep vein thrombosis.

**Table 7:** Candidate values of hyperparameters for LR, RF, XGB, and CatBoost

Model	Parameter Name	Parameter meaning	Candidate values
LR	*C*	regularization strength	0.01, 0.1, 1, 10
	*penalty*	regularization type	l1, l2
RF	*n_estimators*	number of base learners (decision trees)	50, 100, 150, 200
	*max_depth*	maximum depth of a single decision tree	3, 4, 5, 6
	*min_samples_leaf*	minimum number of samples required for a leaf node	5, 10, 15, 20
	*min_samples_split*	minimum number of samples required for node splitting	10, 20, 30
	*max_features*	maximum number of features considered for splitting	sqrt, log2, 0.5
	*bootstrap*	whether to use Bootstrap sampling	True, False
XGBoost	*n_estimators*	number of base learners (decision trees)	50, 100, 150, 200
	*learning_rate*	weight decay coefficient per tree	0.01, 0.05, 0.1, 0.2
	*max_depth*	maximum depth of a single decision tree	3, 4, 5, 6
	*subsample*	sampling ratio of training samples	0.6, 0.8, 1.0
	*colsample_bytree*	feature sampling ratio for constructing each tree	0.6, 0.8, 1.0
	*gamma*	minimum loss reduction required for node splitting	0, 0.1, 0.2
CatBoost	*iterations*	number of base learners (decision trees)	50, 100, 150, 200
	*learning_rate*	weight decay coefficient per tree	0.01, 0.05, 0.1, 0.2
	*depth*	maximum depth of a single decision tree	3, 4, 5, 6
	*l2_leaf_reg*	L2 regularization strength for leaf nodes	1, 3
	*subsample*	sampling ratio of training samples	0.6, 0.8, 1.0
	*min_data_in_leaf*	minimum number of samples required for a leaf node	5, 10, 15, 20
	*bootstrap_type*	sampling strategy type	Bernoulli, MVS

### The superior performance of GBDT models and TabPFN

Our study reaffirms the formidable strength of GBDT models, such as CatBoost and XGBoost, in handling structured tabular data. This finding aligns with the conclusions of numerous previous clinical prediction studies.^27^^,^^28^ By ensembling a multitude of weak learners, these models can effectively capture non-linear relationships and complex interactions among features, while also demonstrating good robustness to noise and missing values in the data. CatBoost, in particular, excelled on several datasets, a success that may be attributed to its unique symmetric tree growth strategy and its advanced algorithms for handling categorical features. These characteristics likely make it especially effective on clinical datasets with higher dimensionality and a greater prevalence of categorical variables.

Even more compelling was the exceptional performance of TabPFN. As a model based on the Transformer architecture and leveraging the “Prior-Data Fitted Network” concept, TabPFN was found to be among the top performers on nearly all datasets in terms of both discrimination and calibration. Several factors may contribute to its success. First, it learns a general-purpose prior through meta-learning on a vast number of synthetic datasets. This enables it to converge quickly and achieve strong performance on new, relatively small datasets (as is common in clinical research) without requiring extensive hyperparameter tuning. Second, its Transformer architecture allows it to effectively capture inter-feature relationships. Our results suggest that TabPFN stands as a potent challenger to traditional GBDT models, especially in scenarios where high performance is desired but computational resources or time for tuning are limited.

### The instability of tabular DL models

In recent years, the academic community has held high expectations from DL models specifically designed for tabular data, anticipating that these models could surpass traditional methods, much as they have in the image and text domains. Our study, however, offers a more circumspect perspective on this view. With the notable exception of TabPFN, several prominent DL models, including FTTransformer, TabNet, and GANDALF, demonstrated considerable instability on our real-world data. For instance, TabNet’s performance on Dataset 1 was worse than random chance, and the performance of FTTransformer varied substantially across different datasets.

This instability likely stems from three key factors. First, DL models typically require a critical mass of data to generalize effectively, which exceeds the sample sizes available in many clinical studies (*N* < 2,000). Second, unlike GBDTs, DL models are highly sensitive to hyperparameters. This high sensitivity explains our rationale for initially evaluating these models, using default or literature-recommended configurations, and reflects the realistic “out-of-the-box” experience for most clinical researchers. As demonstrated in our supplementary experiments (detailed in Methods S2 and Data S3), systematic hyperparameter tuning for DL models required approximately 24 h of CPU computation for just two small datasets. While extensive architecture search might yield improvements, such computational demands are often prohibitive for routine clinical modeling. In practical settings where GPU infrastructure or dedicated computational time is limited, models that achieve robust performance with minimal tuning, such as CatBoost, TabPFN, or AutoGluon, present a clear practical advantage over those requiring exhaustive optimization. Third, DL architectures thrive on unstructured data (images or text) or multimodal inputs. On purely structured tabular data, however, they often struggle to match the efficient inductive bias of decision trees (or the prior-data adaptation of TabPFN) without massive-scale pre-training.

This finding serves as a caution that emerging DL models must undergo rigorous and thorough validation on relevant data before being applied to critical clinical decision-making tasks; one cannot blindly trust performance reported on standardized public benchmarks.

### AutoGluon: A triumph of pragmatism

The robust performance of AutoGluon in this study highlights the immense practical value of AutoML in clinical research. Rather than relying on a single “silver bullet” model, AutoGluon employs sophisticated ensembling and stacking strategies to combine the strengths of multiple diverse models (including GBDTs and neural networks), thereby achieving powerful and stable predictive performance. Our statistical analysis revealed that AutoGluon’s performance was not significantly different from that of the top-performing models (such as TabPFN or CatBoost) on several datasets, and it was almost always significantly superior to the underperforming DL models. For clinical researchers who may lack specialized expertise in ML, AutoGluon provides a reliable, efficient, and user-friendly toolkit. It enables the construction of prediction models with performance that meets or approaches expert-level standards, all with minimal manual intervention.

### Clinical utility and the model selection trade-off

A key finding of this study is that no single model consistently outperformed all others across the full spectrum of clinical utility metrics (PPV, NPV, sensitivity, and specificity). For example, on Dataset 6 and Dataset 8, we observed an extreme trade-off between sensitivity and specificity. Some models, like LR, achieved exceptionally high sensitivity but at the cost of very low specificity, meaning the model could identify nearly all patients but would generate a large number of false alarms. Other models, such as XGBoost, exhibited the opposite behavior. This phenomenon underscores that model selection must be driven by the specific clinical decision. To bridge predictive performance with clinical implementation, decision curve analysis (DCA) provides a practical framework (detailed in Data S1). Clinicians can interpret DCA to select operational thresholds based on the clinical consequences of false positives versus false negatives.

(1) Screening applications (low thresholds, e.g., <0.3). For initial screening of a high-mortality condition (e.g., Dataset 6, IAA), a model with the highest possible sensitivity is required. Clinicians should select a low threshold because the cost of a missed diagnosis is catastrophic. Models exhibiting high net benefit in this lower range (such as baseline LR or CatBoost) are preferable, justifying a higher rate of false alarms that can be clarified with further testing.
(2) Diagnostic confirmation (intermediate thresholds, 0.3–0.7). For standard interventions, clinicians must balance sensitivity and specificity. Top-tier models like TabPFN and CatBoost consistently showed the highest net benefit in this range across our datasets.
(3) Treatment planning (high thresholds, e.g., >0.7). In contrast, for a treatment-initiation decision, such as prescribing potentially toxic drugs or performing invasive procedures, clinicians require a high degree of certainty. At these high thresholds, models maintaining positive net benefit by prioritizing high PPV (such as TabPFN) should be selected to ensure that a high-risk prediction is reliable, thus avoiding unnecessary patient harm.

### Future directions

Future work could proceed in several directions. First, to establish a more robust evidence base and confirm broader applicability across diverse clinical environments, this study must be replicated on larger-scale, multicenter datasets. Second, future research could explore optimal preprocessing and feature engineering strategies tailored to different model architectures. Finally, future work must move beyond aggregate performance metrics to rigorously assess clinical trustworthiness and generalizability. This requires a multifaceted approach: (1) Subgroup and fairness audits: analyses must be conducted to ensure models perform reliably and equitably across clinically relevant subgroups and to identify and mitigate any potential demographic biases. (2) Explainability and plausibility: a crucial next step is to integrate explainable AI techniques, such as SHAP (SHapley Additive exPlanations), to analyze the decision-making logic of high-performing models like TabPFN and CatBoost. This is essential to verify that their predictions are driven by known clinical risk factors and not by spurious correlations, which is a prerequisite for clinical adoption.

While this study focused on specialized tabular models, the rise of LLMs offers new avenues for clinical prediction. Recent research suggests that while general-purpose LLMs excel at interpreting unstructured medical notes, they currently lag behind specialized models like XGBoost and TabPFN in processing pure numerical tabular data for precise risk scoring. However, a promising future direction is the fusion of LLMs with tabular models, using LLMs to extract semantic features from medical text and feeding them into robust tabular solvers like TabPFN to enhance predictive performance.

Through a large-scale benchmark evaluation on eight real-world clinical datasets, this study systematically compared the performance of classic ML, tabular DL, and AutoML paradigms for clinical risk prediction. Our findings clearly indicate that GBDT models, particularly CatBoost, and the emerging tabular DL model TabPFN, collectively constitute the current top tier of performance. However, model selection must align with the specific clinical objective. While TabPFN and CatBoost demonstrated superior overall discrimination (high AUC/AP) suitable for confirmatory diagnostics, baseline models like LR occasionally exhibited higher sensitivity, making them potentially useful for initial screening tasks where minimizing false negatives is the priority.

At the same time, our findings offer a circumspect perspective on the current enthusiasm for tabular DL. With the notable exception of TabPFN, most state-of-the-art DL models exhibited significant instability on real-world clinical data, with their performance being highly dependent on dataset characteristics. This suggests that considerable caution should be exercised when deploying such models in clinical practice. Furthermore, the AutoML framework AutoGluon, powered by its strong ensembling capabilities, proved to be an efficient, reliable, and accessible solution, offering a practical shortcut for clinical researchers to build high-performance predictive models.

In summary, within the context of the evaluated single-center cohorts, this study provides solid empirical evidence and practical guidance for clinical data scientists and researchers, aiding them in making informed trade-offs and selecting the most appropriate modeling strategy for specific risk prediction tasks.

### Limitations of the study

The primary strengths of this study are 3-fold: (1) We used a diverse set of eight uncurated datasets from a real-world clinical environment, rather than sanitized public benchmarks, which enhances the real-world relevance and generalizability of our conclusions. (2) The scope of comparison was extensive, marking one of the first studies to systematically compare classic ML, state-of-the-art tabular DL, and AutoML paradigms within a unified framework. (3) We employed a rigorous cross-validation and performance evaluation protocol, ensuring the reliability of our results.

Nevertheless, this study is subject to several limitations: (1) Single-center origin and sample size. All data were sourced from a single hospital system. While we intentionally focused on “small-to-medium”-sized datasets (*N* < 2,000) to reflect typical clinical research scenarios, rather than utilizing massive public benchmarks like MIMIC-IV, this implies that our data inherently reflect institution-specific factors, such as regional patient demographics, local clinical protocols, and data entry workflows. Consequently, while our findings regarding the superiority of TabPFN and CatBoost are robust within this context, they should not be assumed to be universally applicable without external validation to account for potential distributional shifts across different institutions. To address this concern, we have provided supplementary benchmarking on the publicly accessible Pima Indians Diabetes Database (detailed in Methods S1 and Data S2). While the consistent performance hierarchy observed on this independent public dataset partially mitigates this concern, we must remain cautious regarding the broader applicability of our findings. The inherent biases of a single-institution cohort mean that our conclusions cannot be definitively generalized to all clinical environments. Future studies incorporating large-scale, multicenter datasets are essential to strengthen the evidence base. (2) We applied a uniform preprocessing pipeline to all models, which may not have allowed certain models, particularly the DL architectures, to perform to their full potential. (3) In the primary analysis, DL models were evaluated with default parameters due to the significant computational constraints inherent to their complex architectures. Although this setup might not capture the absolute performance ceiling of these models, it represents the typical resource limits in clinical research. We addressed this limitation through supplementary systematic hyperparameter tuning on Dataset 1 and Dataset 2 (detailed in Methods S2 and Data S3). These supplementary results confirmed that while tuning yielded modest gains for certain DL models, it did not alter the fundamental performance hierarchy, further underscoring the practical superiority of models that are less dependent on intensive tuning. (4) This study primarily focused on predictive performance metrics and did not conduct comprehensive fairness, subgroup, and interpretability analyses. While not strictly required for an initial benchmarking evaluation, the absence of these analyses remains a notable limitation, as these aspects are absolutely the prerequisites for the safe, transparent, and equitable clinical deployment of these models. Finally, we did not specifically analyze the influence of sex, age, or patient demographics on model performance, which must be rigorously evaluated in future studies prior to clinical deployment.

## Resource availability

### Lead contact

Further information and requests for resources and reagents should be directed to and will be fulfilled by the lead contact, Sujuan Tang, Email: tangsujuan108@163.com.

### Materials availability

This study did not generate new unique reagents.

### Data and code availability

• Data: The datasets generated and analyzed during the current study are not publicly available due to ethical and privacy restrictions. However, data may be made available to qualified researchers upon reasonable request to the corresponding author, subject to a formal data use agreement and approval from the Institutional Review Board of the Affiliated Hospital of Jining Medical University. Supplementary benchmarking results on the publicly available Pima Indians Diabetes Database can be accessed at https://www.kaggle.com/datasets/uciml/pima-indians-diabetes-database/data.
• Code: The complete source code used for model training and evaluation has been deposited at GitHub and is publicly available as of the date of publication at: https://github.com/QingwWu/TabularML.
• Other: Any additional information required to reanalyze the data reported in this paper is available from the lead contact upon request.

## Acknowledgments

The study was supported by PhD Research Foundation of Affiliated Hospital of 10.13039/501100008853Jining Medical University (grant no. 2024-BS-009) and Clinical Research Support Program of the Affiliated Hospital of 10.13039/501100008853Jining Medical University (Clinical Medical College) (grant no. LCYJ-020).

## Author contributions

Q.W. was responsible for the modeling experiments, formal analysis, and implementation of the algorithms. S.T., Z.Q., Q.L., and C.H. contributed to data collection and data curation and provided critical clinical support for the study; S.T. also took the lead in drafting and preparing the manuscript. All authors have reviewed, edited, and approved the final version of the manuscript.

## Declaration of interests

The authors declare that they have no known competing financial interests or personal relationships that could have appeared to influence the work reported in this paper.

## Declaration of generative AI and AI-assisted technologies in the writing process

During the preparation of this work, the author(s) used Kimi in order to improve readability and language. After using this tool/service, the authors reviewed and edited the content as needed and take full responsibility for the content of the published article.

## STAR★Methods

### Key resources table

**Table:** 

REAGENT or RESOURCE	SOURCE	IDENTIFIER
**Deposited data**		

Eight anonymized clinical risk prediction datasets	Affiliated Hospital of Jining Medical University	N/A (Data sharing subject to formal Data Use Agreement)
Pima Indians Diabetes Database	Kaggle/UCI Machine Learning Repository	https://www.kaggle.com/datasets/uciml/pima-indians-diabetes-database/data

**Software and algorithms**		

Python (v3.10)	Python Software Foundation	https://www.python.org/
scikit-learn (v1.6.0)	scikit-learn developers	https://scikit-learn.org/
xgboost (v3.0.4)	XGBoost developers	https://xgboost.readthedocs.io/
catboost (v1.2.8)	Yandex	https://catboost.ai/
PyTorch-Tabular (v1.1.1)	PyTorch-Tabular developers	https://github.com/manujosephv/pytorch_tabular
tabpfn (v2.13)	TabPFN developers	https://github.com/automl/TabPFN
autogluon.tabular (v1.4.0)	AutoGluon developers	https://auto.gluon.ai/
scipy (v1.16.1)	SciPy developers	https://scipy.org/
numpy (v1.26.4)	NumPy developers	https://numpy.org/
Custom code for model training and evaluation	This paper	https://github.com/QingwWu/TabularML

### Experimental model and study participant details

This study utilized eight anonymized clinical risk prediction datasets obtained from the Affiliated Hospital of Jining Medical University in China. These datasets were derived from real-world clinical practice between 2019 and 2025. The datasets comprise anonymized retrospective patient records. Detailed demographic breakdowns (e.g., age, sex, ethnicity) varied by dataset and were not the primary focus of this methodological benchmarking study. The collection and use of all data were approved by the Institutional Review Board of the the Affiliated Hospital of Jining Medical University (Approval No: 2025-03-C025), and the requirement for patient informed consent was waived.

Each dataset represents a distinct clinical prediction task. To comprehensively evaluate the generalization capabilities of the models, we selected datasets that exhibit diversity in sample size, number of features, and event rates. Detailed statistical characteristics are summarized in Table 6. To ensure reproducibility and clarity, the detailed clinical definitions of outcomes and the specific lists of variables for each dataset are provided in Data S4.

### Method details

#### Data preprocessing

To ensure fairness and stability during model training, a uniform preprocessing pipeline was applied to all datasets.

(1) Missing Value Imputation: Numerical features were imputed using the median, while categorical features were imputed using the mode.
(2) Feature Encoding: One-hot encoding was applied to all categorical features.
(3) Feature Scaling: Standardization was applied to all numerical features to scale them to a mean of 0 and a standard deviation of 1.

To prevent data leakage, all preprocessing steps were performed independently within the training fold of each cross-validation split.

#### Model selection and implementation

This study compared a total of 10 models across three distinct categories.

(1) Classic Machine Learning Models. Logistic Regression (LR), a widely recognized baseline model for clinical prediction, implemented using *scikit-learn* library(v1.6.0); Random Forest (RF), a decision-tree-based ensemble model, implemented using *scikit-learn* library(v1.6.0); XGBoost (XGB), an efficient implementation of gradient boosting decision trees, implemented using *xgboost* library(v3.0.4); CatBoost, a gradient boosting framework particularly adept at handling categorical features, implemented using *catboost* library(v1.2.8).
(2) Tabular Deep Learning Models. TabNet, FTTransformer (FTTrans), CategoryEmbedding (Category), and GANDALF were implemented via *PyTorch-Tabular* library(v1.1.1), a library that provides a unified interface for mainstream tabular deep learning models. TabPFN was implemented using the *tabpfn* library (v2.13). For these models, we adopted the default architectural and parameter settings provided by their respective libraries.
(3) Automated Machine Learning (AutoML) Framework. AutoGluon, implemented using *autogluon.tabular* library(v1.4.0). The framework was configured for '*best quality*’ (presets = 'best_quality') and was set to automatically perform model selection, hyperparameter tuning, and ensembling within a specified time limit (time_limit = 600 s).

#### Experimental design and hyperparameter tuning

To obtain robust and unbiased performance estimates, we employed a 10-times repeated 5-fold cross-validation strategy. The procedure was as follows.

(1) Each dataset was randomly shuffled and partitioned into five mutually exclusive subsets (folds).
(2) In each iteration, 4-folds were used for training, and the remaining fold was used for testing. This process was repeated five times, ensuring that each fold served as the test set once.
(3) The entire 5-fold cross-validation process was repeated 10 times with different random shuffles. This resulted in 50 performance evaluations (5-folds × 10 repetitions) for each model on each dataset.

For the classic machine learning models (LR, RF, XGBoost, CatBoost), we performed a grid search to identify the optimal hyperparameter combination on the training set of each fold. The search space covered key parameters such as learning rate, number of trees, and tree depth (detailed in Table 7). Due to their higher computational cost, the deep learning models were evaluated using their default, literature-recommended parameter configurations. AutoGluon automatically executed its internal hyperparameter optimization process. However, to ensure a balanced comparison, we conducted systematic hyperparameter tuning for all deep learning models (FTTrans, TabNet, GANDALF, Category) on Datasets 1 and 2 using grid search over architecture-specific parameter spaces (detailed in Methods S2 and Data S3). These supplementary experiments confirmed that while parameter optimization improved performance for some deep learning models, it did not substantially alter the relative performance hierarchy observed with default parameters.

### Quantification and statistical analysis

#### Performance evaluation metrics

We assessed model performance from three perspectives: discrimination, calibration, and clinical utility.

(1) Discrimination refers to the model’s ability to distinguish between positive and negative cases. Area Under the Receiver Operating Characteristic (ROC) Curve (AUC) was the primary metric for evaluating discrimination. Area Under the Precision-Recall (PR) Curve (AP) serves as a critical supplement to AUC, especially for datasets with class imbalance.
(2) Calibration measures the agreement between a model’s predicted probabilities and the observed event frequencies. Calibration Curve was generated by binning predicted probabilities and plotting the mean predicted probability against the actual proportion of positive cases in each bin. Ideally, the curve should align with the diagonal line. Brier Score, calculated as the mean squared error between the predicted probabilities and the actual outcomes. A lower Brier score indicates better calibration.
(3) Clinical Utility Metrics. An optimal diagnostic threshold was determined for each model by maximizing the Youden’s index on the ROC curve. At this threshold, we calculated sensitivity, specificity, positive predictive value (PPV), and negative predictive value (NPV).

Additionally, Decision Curve Analysis (DCA) was performed to assess net clinical benefit across threshold probabilities. Detailed DCA results are provided in Data S1.

#### Statistical analysis

All performance metrics are reported as the mean and 95% confidence interval (CI) derived from the 10-times repeated 5-fold cross-validation. We acknowledge that while this repeated protocol stabilizes the mean performance estimates for small datasets, the resulting confidence intervals may be slightly optimistic due to the overlap of data across repetitions. To determine whether the performance differences between models were statistically significant, we performed pairwise comparisons of AUC values using the Wilcoxon signed-rank test with Bonferroni correction. A *p*-value of less than 0.01 was considered to indicate a statistically significant difference. All statistical analyses were conducted using Python (v3.10) and relevant scientific computing libraries, including *scipy* (v1.16.1) and *numpy* (v1.26.4).

## Supplemental information

Document S1. Figures S1–S8, Data S1–S3, and Methods S1 and S2

Data S4. Supplemental notes on variables for the eight datasets, related to Table 6
