# Supplementary material for: Systematic evaluation of machine learning models for clinical risk prediction on real-world hospital datasets
Source: iScience. 2026 Apr 8;29(5):115654. doi: 10.1016/j.isci.2026.115654 (PMC13127387; doi:10.1016/j.isci.2026.115654)
Supplement: Document S1. Figures S1–S8, Data S1–S3, and Methods S1 and S2 [file mmc1.pdf]

iScience, Volume *29*

Supplemental information

Systematic evaluation of machine learning models for clinical risk prediction on real-world hospital datasets

Qingwen Wu, Ziyou Qi, Qingwei Li, Cuiping Hao, and Sujuan Tang

Data S1: Decision Curve Analysis

Figure S1–S8: Decision Curve Analysis for Datasets 1–8

Reference Lines: "Treat None" (dashed black): Net benefit = 0 (horizontal line); "Treat All" (dotted gray): Net benefit varies by disease prevalence

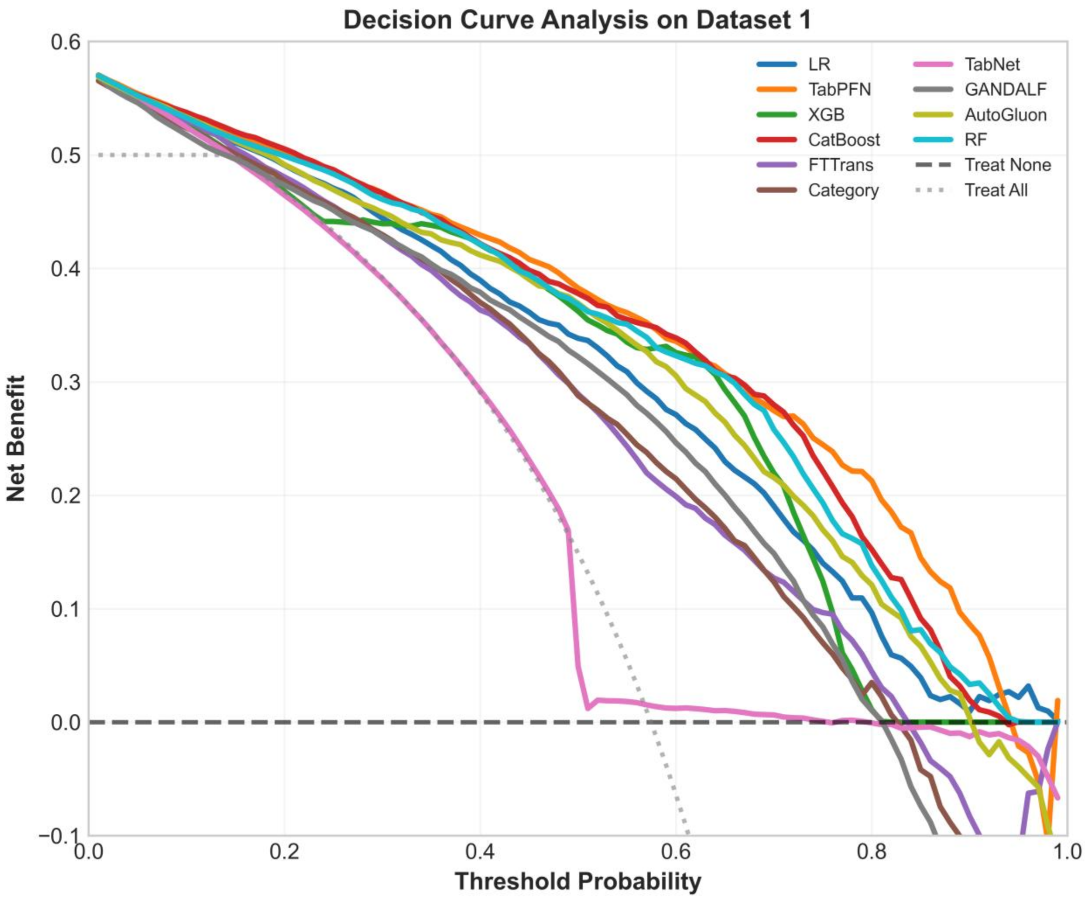

Figure S1: Dataset 1

Key Findings: **TabPFN** (orange) and **CatBoost** (red) maintain positive net benefit across the broadest threshold range (0.1-0.9); TabNet (pink) falls below "Treat None" at thresholds>0.4, indicating clinical harm; Optimal clinical utility for top models: threshold range 0.3-0.7; Classic ML models (LR, RF, XGB) show competitive performance with stable net benefit curves.

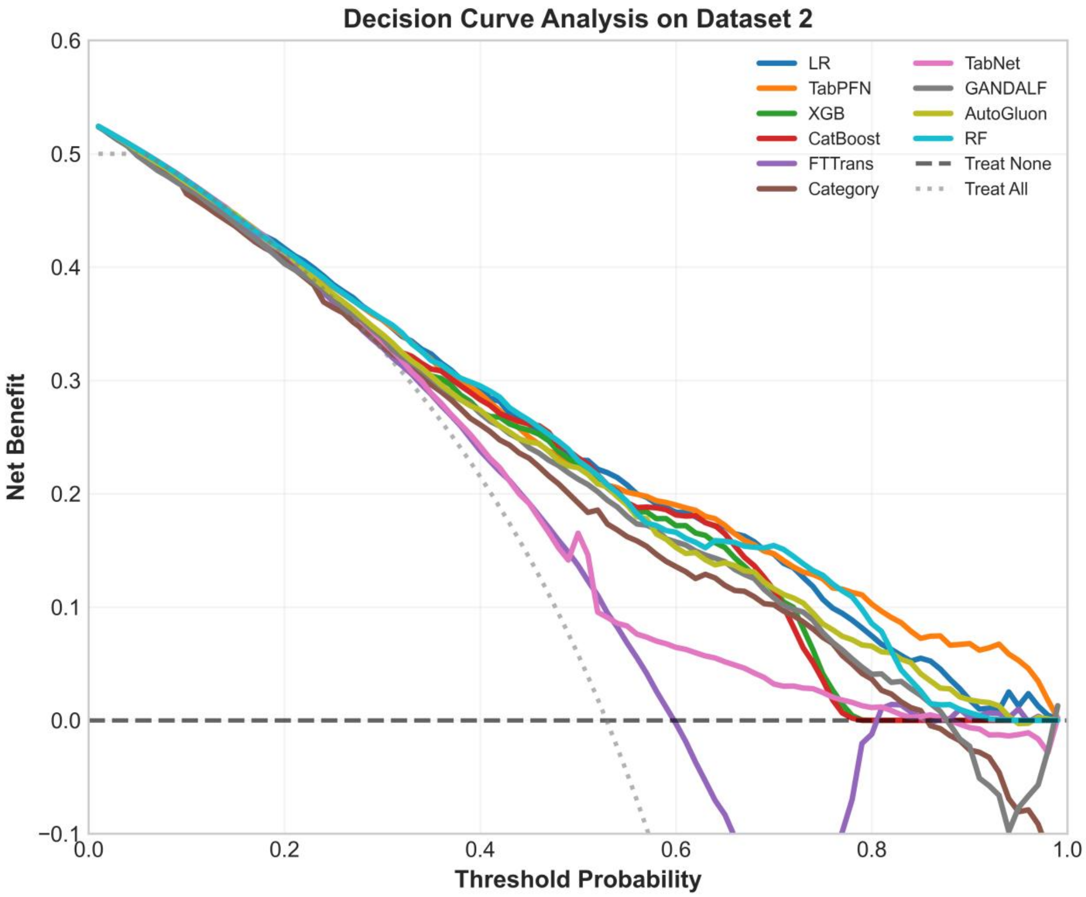

Figure S2: Dataset 2

Key Findings: All models show reduced net benefit compared to Dataset 1, reflecting lower discriminative performance; TabPFN maintains slight advantage at thresholds 0.2-0.6; FTTransformer (purple) shows negative net benefit across most thresholds; Top-tier models (TabPFN, CatBoost, LR) cluster closely together.

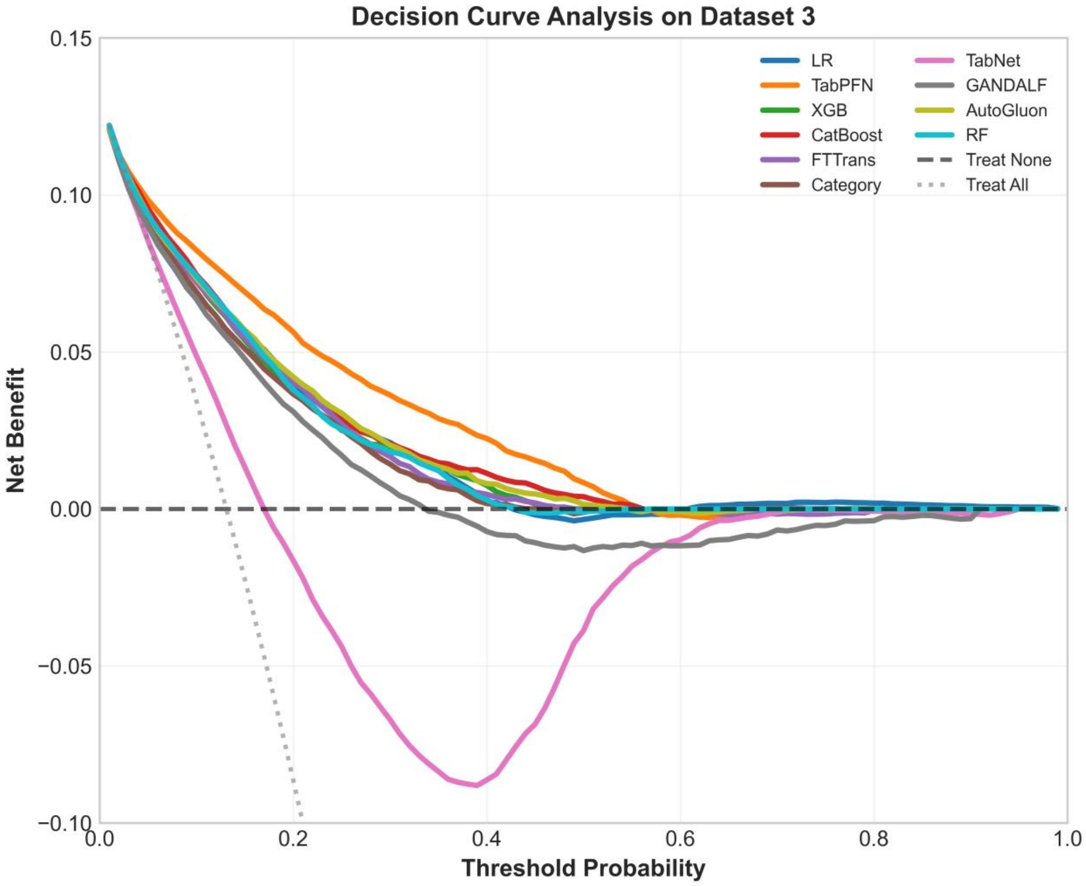

Figure S3: Dataset 3

Key Findings: Low prevalence results in compressed net benefit scale (max ~0.12); TabPFN dominates at thresholds 0.05-0.3, critical for screening; Most models converge to zero benefit at thresholds>0.5; GANDALF (gray) shows negative net benefit at intermediate thresholds; TabNet exhibits extreme instability with deep negative net benefit at thresholds 0.3–0.5.

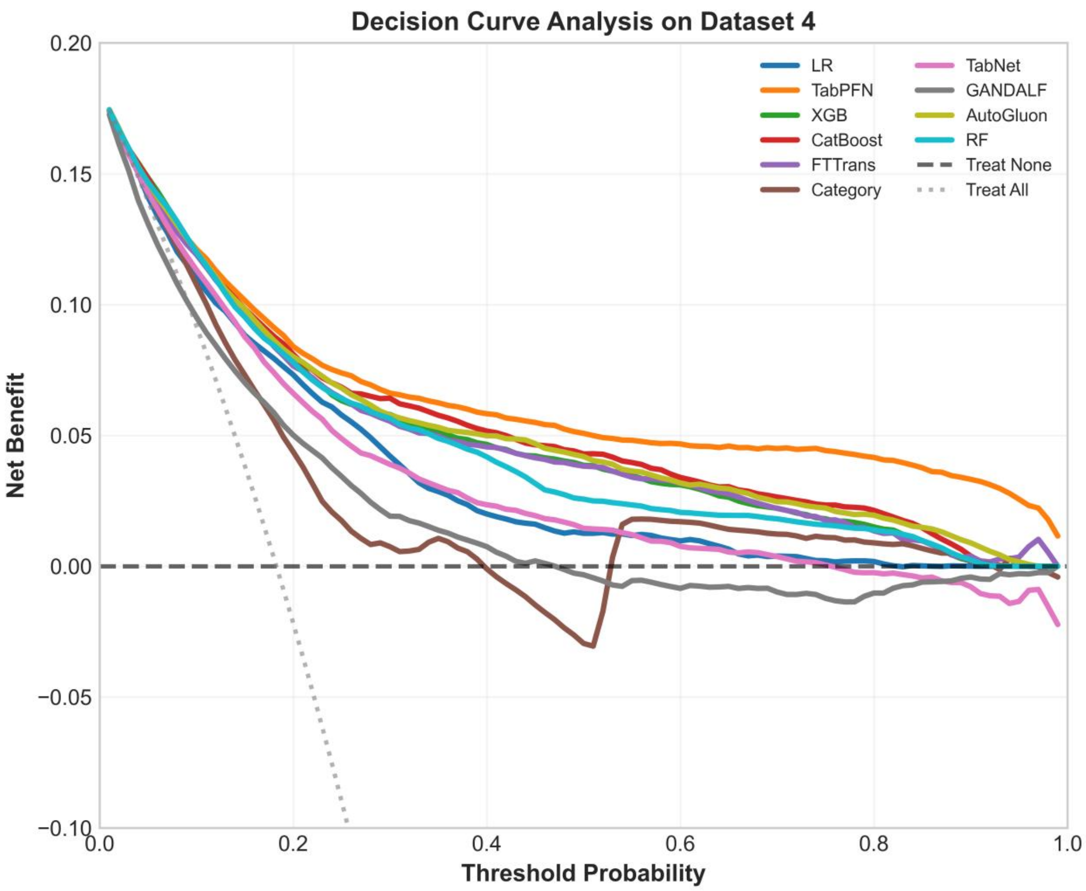

Figure S4: Dataset 4

Key Findings: Higher prevalence than Dataset 3 enables broader useful threshold range; TabPFN, CatBoost, AutoGluon, and XGBoost form top tier with similar net benefit profiles; Clear separation between gradient boosting/TabPFN and other deep learning models; Category (brown) shows erratic behavior with negative net benefit at thresholds 0.4-0.5.

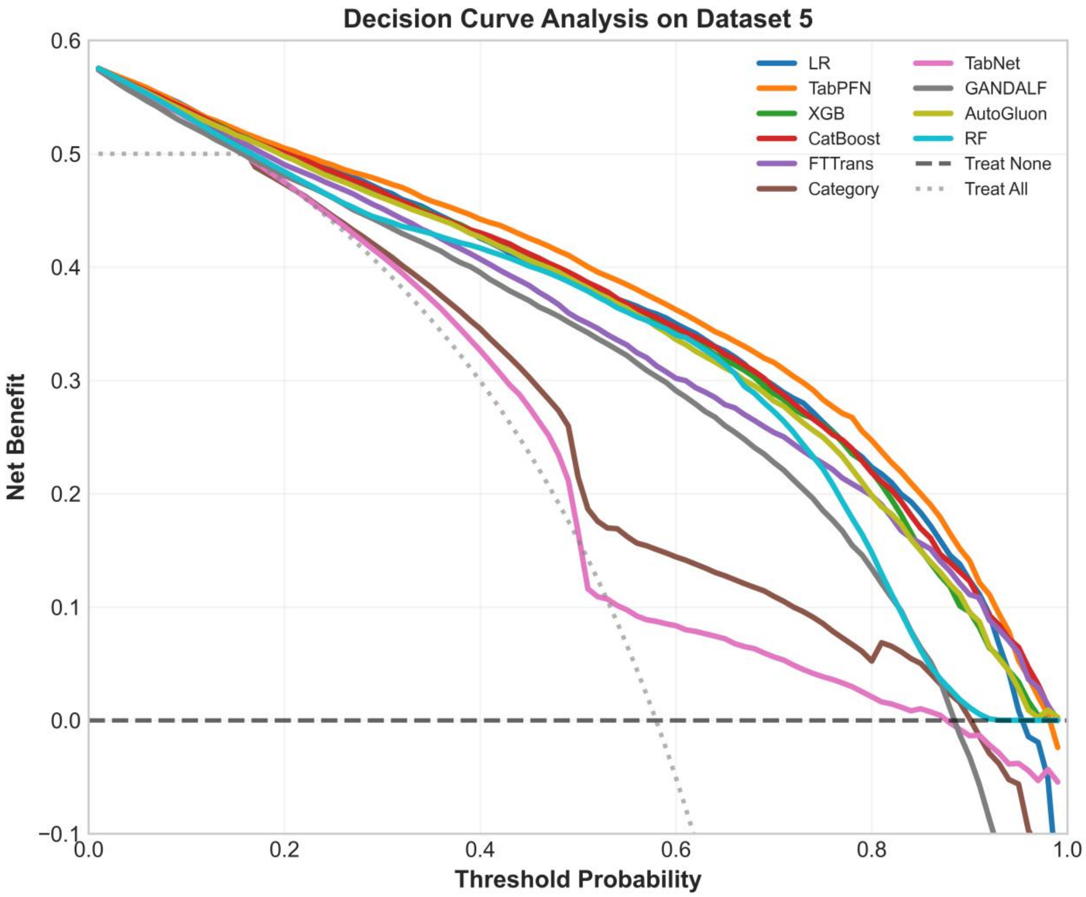

Figure S5: Dataset 5

Key Findings: Broad positive net benefit range (0.1-0.9) for top models due to balanced classes; TabPFN maintains consistent advantage across nearly all thresholds; TabNet and Category show early decline in net benefit (thresholds>0.4); Classic ML models perform competitively with deep learning top performers.

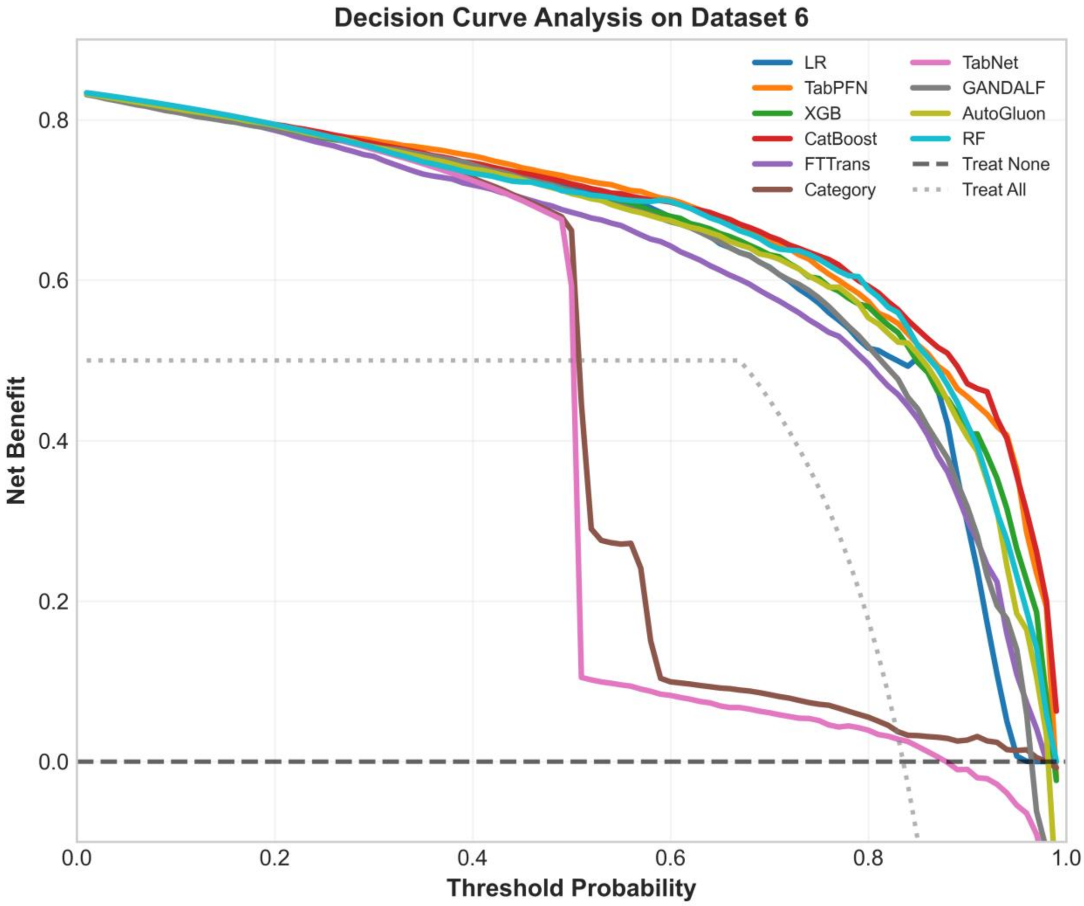

Figure S6: Dataset 6

Key Findings: High prevalence shifts optimal strategy toward "Treat All" at low thresholds; Step-like DCA curves reflect highly discriminative features in this dataset; CatBoost and RF achieve highest net benefit at clinical thresholds (0.2-0.6); All models converge near zero benefit at high thresholds (>0.8) due to high baseline risk; TabNet and Category show dramatic drops in net benefit at threshold ~0.5.

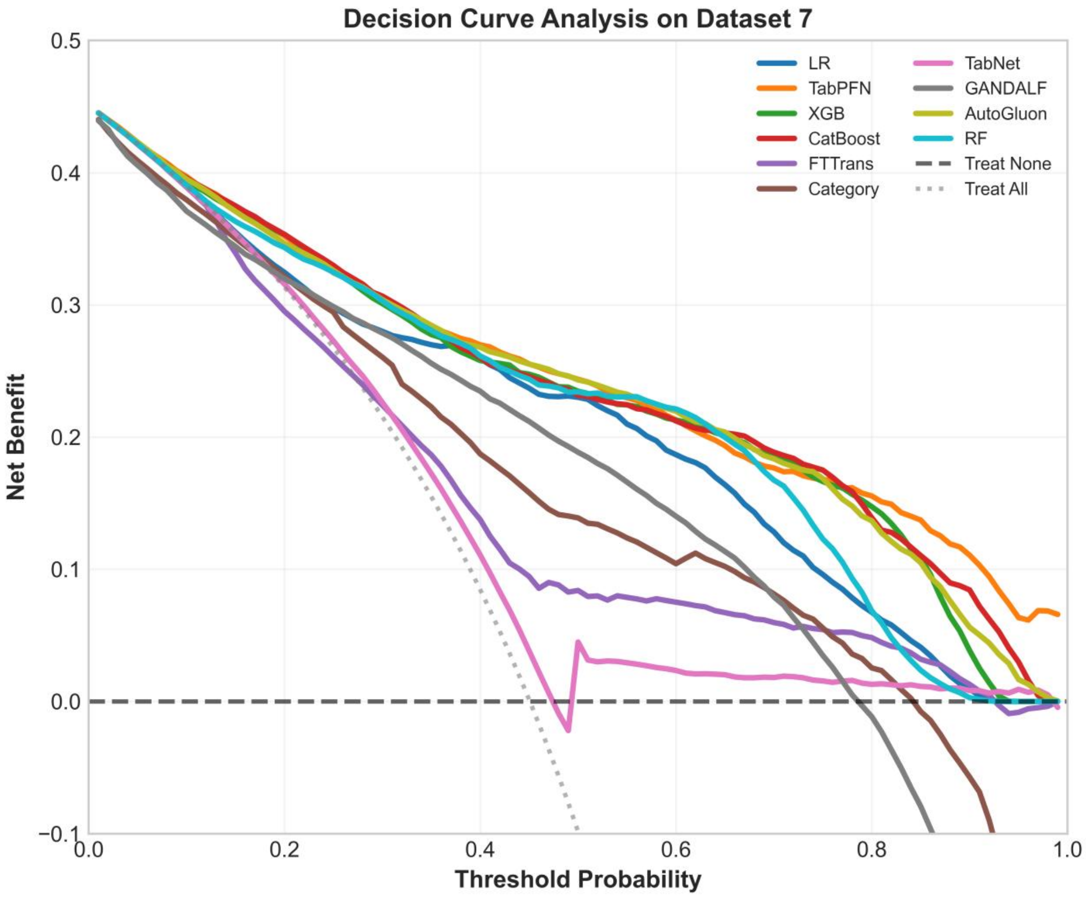

Figure S7: Dataset 7

Key Findings: TabPFN, AutoGluon, CatBoost, and RF show comparable net benefit profiles; Broad useful range (0.2-0.8) for top-tier models; FTTransformer and TabNet demonstrate unstable, lower net benefit; GANDALF shows negative net benefit at high thresholds (>0.8)

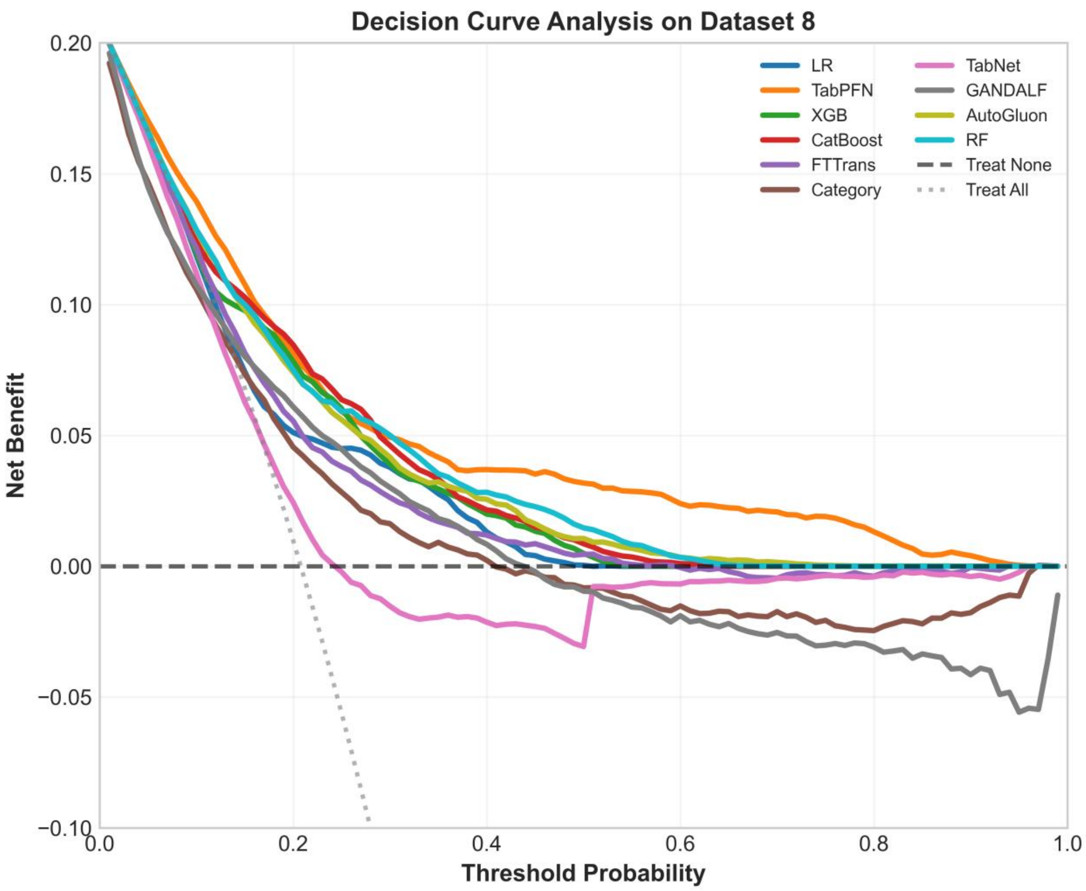

Figure S8: Dataset 8

Key Findings: Most challenging prediction task with lowest overall net benefits; TabPFN maintains relative advantage at thresholds 0.1-0.5; Narrow window of clinical utility for all models due to difficulty of DVT prediction in ICU; GANDALF and Category show negative net benefit at multiple thresholds; TabNet falls below "Treat None" at thresholds >0.3.

Data S2: Performance metrics on Pima Indians Diabetes Database

Model	AUC	ACC	Sensitivity	Specificity	PPV	NPV	F1	Kappa	Youden
**TabPFN**	**0.8407**	0.7656	0.5884	0.8606	0.6935	0.7960	0.6367	0.4654	0.4490
**CatBoost**	0.8335	0.7651	0.5709	0.8692	0.7005	0.7908	0.6291	0.4598	0.4401
RF	0.8298	0.7629	0.5478	0.8782	0.7068	0.7837	0.6172	0.4494	0.4260
LR	0.8283	0.7678	0.5504	0.8844	0.7185	0.7859	0.6233	0.4597	0.4348
AutoGluon	0.8253	0.7612	0.5713	0.8630	0.6909	0.7897	0.6254	0.4524	0.4343
XGB	0.8183	0.7495	0.4660	0.9014	0.7170	0.7590	0.5649	0.3999	0.3674
GANDALF	0.8113	0.7503	0.6000	0.8308	0.6553	0.7949	0.6264	0.4394	0.4308
Category	0.6744	0.6835	0.3761	0.8482	0.5705	0.7172	0.4533	0.2436	0.2243
FTTrans	0.5850	0.6383	0.2593	0.8414	0.4671	0.6794	0.3335	0.1123	0.1007
TabNet	0.4232	0.6020	0.1138	0.8636	0.3090	0.6452	0.1663	-0.0265	-0.0226

Data S3: Performance metrics after hyperparameter optimization

Dataset 1: Cryptogenic Stroke Prediction

Model	AUC	ACC	PPV	NPV	Recall	Speci	Configuration
LR	0.8312	0.7640	0.7751	0.7557	0.8366	0.6659	Grid search
XGB	0.8806	0.7759	0.7710	0.8004	0.8746	0.6427	Grid search
RF	0.8804	0.7818	0.7758	0.8065	0.8795	0.6501	Grid search
CatBoost	0.8832	0.7872	0.7817	0.8094	**0.8805**	0.6614	Grid search
AutoGluon	**0.8869**	0.7914	0.7893	0.8082	0.8771	0.6772	AutoML
TabPFN	0.8640	**0.7920**	0.7870	**0.8108**	0.8802	0.6717	Default
FTTrans	0.8473	0.7733	**0.7911**	0.7592	0.8286	**0.6997**	Optimized↑
TabNet	0.6671	0.6348	0.6849	0.5886	0.6750	0.5827	Optimized↑
GANDALF	0.8332	0.7715	0.7841	0.764	0.8342	0.6872	Optimized↑
Category	0.8258	0.7579	0.7898	0.7325	0.7951	0.7075	Optimized↑
FTTrans	0.7604	0.7148	0.7379	0.6402	0.8255	0.5659	Default
TabNet	0.3496	0.5949	0.6359	0.4025	0.7920	0.3289	Default
GANDALF	0.8142	0.6458	0.6787	0.5197	0.8045	0.4317	Default
Category	0.7772	0.6627	0.6827	0.5578	0.7984	0.4795	Default

Dataset 2: Cardiac Parameter-Based Cryptogenic Stroke Prediction

Model	AUC	ACC	PPV	NPV	Recall	Speci	Note
LR	0.7855	**0.7006**	0.7135	**0.6927**	0.7341	0.6630	Grid search
XGB	0.7742	0.6990	0.7109	0.6926	0.7344	0.6592	Grid search
RF	0.7833	0.6995	0.7140	0.6897	0.7276	0.6678	Grid search
CatBoost	**0.7856**	0.7002	0.7158	0.6892	0.7252	0.6721	Grid search
AutoGluon	0.7845	0.6990	0.7148	0.6876	0.7229	0.6703	AutoML
TabPFN	0.7758	0.6982	0.7155	0.6870	0.7218	0.6734	Default
FTTrans	0.7471	0.6226	0.6524	0.5939	0.4159	**0.8568**	Optimized↑
TabNet	0.6530	0.5548	0.5494	0.6426	0.8413	0.2322	Optimized↓
GANDALF	0.7770	0.7016	**0.7465**	0.6808	0.6914	0.7121	Optimized↑
Category	0.7022	0.6516	0.6656	0.5391	0.7707	0.5155	Optimized↓
FTTrans	0.6761	0.6077	0.6224	0.3378	**0.8470**	0.3390	Default
TabNet	0.7279	0.6219	0.6297	0.5236	0.8325	0.3856	Default
GANDALF	0.7677	0.6426	0.6588	0.5694	0.7812	0.4870	Default
Category	0.7323	0.6477	0.6667	0.5697	0.7619	0.5195	Default

Methods S1: Experimental protocol for Pima Indians Diabetes Database validation

1. Data source: Kaggle repository (https://www.kaggle.com/datasets/uciml/pima-indians-diabetes-database/data), originally sourced from the UCI Machine Learning Repository
2. Sample: 768 female Pima Indian patients, age⩾21
3. Features: 8 clinical variables (pregnancies, glucose, blood pressure, skin thickness, insulin, BMI, pedigree, age)
4. Outcome: Binary diabetes status (268 positive, 500 negative)
5. Validation: 10× repeated 5-fold stratified cross-validation
6. Preprocessing: Median imputation (continuous), mode imputation (categorical)
7. Evaluation metrics: AUC, ACC, Sensitivity, Specificity, PPV, NPV, F1, Kappa, Youden's Index

Methods S2: Hyperparameter optimization protocol for deep learning models

Optimization scope: FTTransformer, TabNet, GANDALF, CategoryEmbedding on Datasets 1-2

Parameter search space:

Model	Parameter	Search Space
**FTTransformer**	num_attn_blocks	[2, 4, 6]
	num_heads	[2, 4, 8]
	attn_dropout	[0.0, 0.1, 0.2]
**TabNet**	n_d	[8, 16, 32]
	n_a	[8, 16, 32]
	n_steps	[3, 5, 7]
	gamma	[1.0, 1.5, 2.0]
**GANDALF**	gflu_stages	[4, 6, 8, 10]
	gflu_dropout	[0.0, 0.1, 0.2]
**CategoryEmbedding**	layers	["128-64", "256-128-64", "512-256-128"]
	dropout	[0.0, 0.1, 0.2]

Procedure:

1. Grid search over specified parameter ranges

2. 10× repeated 5-fold cross-validation

3. Evaluation metrics: AUC, ACC, PPV, NPV, Recall, Specificity

4. Hardware: Intel Xeon Gold 5418Y, 512GB RAM, CPU-only

5. Time: ~24 hours for Datasets 1-2
